# Indoleamine 2,3-dioxygenase 1 alters the proportions of B cell subpopulations in the microenvironment of acute myeloid leukemia

**DOI:** 10.1186/s43556-025-00262-x

**Published:** 2025-04-16

**Authors:** Yu Yao, Yu-ying Liu, Jian-feng Li, Yun-shuo Chen, Lei Shi, Yang Shen, Li-li Yang, Qing Yang

**Affiliations:** 1 State Key Laboratory of Genetics and Development of Complex Phenotypes, School of Life Sciences, Fudan University, Songhu Road 2005, Shanghai, 200438 China; 2 State Key Laboratory of Medical Genomics, Shanghai Institute of Hematology, National Research Center for Translational Medicine at Shanghai, Ruijin Hospital Affiliated to Shanghai Jiao Tong University School of Medicine, Shanghai, 200025 China; 3 State Key Laboratory of Druggability Evaluation and Systematic Translational Medicine, Tianjin Medical University Cancer Institute and Hospital, National Clinical Research Center for Cancer, Tianjin, 300060 China

**Keywords:** Acute myeloid leukemia (AML), Immunosuppression, Indoleamine 2,3-dioxygenase 1 (IDO1), B cells, IDO1 inhibitors

## Abstract

Acute myeloid leukemia (AML), the most common leukemia in adults, exhibits immune escape characteristics like solid tumors. The expression of indoleamine 2,3-dioxygenase 1 (IDO1), a well-recognized immune checkpoint, has been detected in AML blast cells and is associated with poor clinical outcome. Although an imbalance of B cell subpopulations exists in AML patients’ bone marrow microenvironment, the role of B cells and their interaction with IDO1 in AML have yet to be elucidated. Herein, with bioinformatic analysis, we found the close correlations between IDO1 expression and survival and B cell subpopulation proportions in AML patients. Further, our investigation into IDO1 expression and activity, B cell subpopulation proportions and immunosuppressive interleukin-10 (IL-10) level in AML cells and clinical samples revealed significant findings. Using a co-culture system of healthy human PBMCs and AML cell lines, we demonstrated that high IDO1 expression in AML cells could alter the proportions of total B, regulatory B and memory B cells, and increased the level of IL-10. Finally, with the IDO1 inhibitor RY103 designed by our laboratory, we found that IDO1 inhibition had good anti-leukemic effect and restored the abnormal proportions of B cell subpopulations in AML mice. Our study is the first to reveal the modulation of IDO1 on B cell subpopulations in AML, making a significant breakthrough in understanding the immune escape mechanisms of AML. Application of IDO1 inhibitor, such as RY103, targeting the imbalance of B cell subpopulations can lead to innovative treatments for AML.

**Supplementary Information:**

The online version contains supplementary material available at 10.1186/s43556-025-00262-x.

## Introduction

Acute myeloid leukemia (AML), the most prevalent leukemia in adults, is marked by abnormal proliferation of leukemic blasts in both bone marrow (BM) and peripheral blood (PB). Nevertheless, the underlying immunological mechanism of AML is not yet fulled elaborated [1–3]. AML shares similar immune evasion mechanisms with solid tumors, including the upregulation of amino acid metabolic enzymes such as indoleamine 2,3-dioxygenase-1 (IDO1) [4] and arginase-2 (ARG2) [5], downregulation of antigen presentation molecules, overexpression of inhibitory T cell ligands, and the production of reactive oxygen species. Thus, in turn, dysfunction of natural killer (NK) cells and T cells [6], proliferation and activation of immunosuppressive cell populations like regulatory T cells (Tregs) [7], myeloid-derived suppressor cells (MDSCs) [8] and tumor-associated macrophages (TAMs) [9] can be induced.

IDO1, an immunomodulatory enzyme, catabolizes the degradation of tryptophan (Trp) into N-formyl-kynurenine, which represents the intial and rate-limiting step of the kynurenine pathway (KP) [10]. IDO1 has emerged as a key player in suppressing anti-tumor responses and fostering tumor immune evasion [11].The immunosuppressive effect of IDO1 is driven by the depletion of Trp and the accumulation of Kyn, that affects immune cells such as NK cells [12, 13], effector T cells [14], Tregs [15], tolerant dendritic cells (DCs) [16], and MDSCs [17], leading to tumor immune evasion [18]. Several IDO1 selective inhibitors have been developed. IDO1 inhibitory activities and anti-tumor effects of epacadostat [19, 20], 1-methyl-L-tryptophan (1-L-MT) [21, 22] and RY103 [23–25] in CT26 colorectal cancer, KPIC pancreatic carcinoma, GL261 glioma and B16F10 melanoma mouse models have been demonstrated. It has been reported that IDO1 expresses in both BM and PB AML blasts [26–29], whose expression negatively correlates with clinical outcome, including the response to chemotherapy, risk of relapse and overall survival (OS) [30–33]. IDO1 constitutively expressing AML cells co-cultured with T cells and DCs can inhibit CD4^+^and CD8^+^T cell proliferation, promote Tregs proliferation, impair DCs maturation, and eventually suppress anti-tumor immune response [34–36].Despite the fact that 3 clinical studies (NCT03491579, NCT03444649, NCT02835729) have been conducted to explore the efficacy of combination of epacadostat and chemotherapy in AML [11], the role of IDO1 in AML and its underlying mechanism is still uncertain.

There is limited research on the potential contribution of B cells to the anti-tumor immune response during tumor progression [9]. Despite the growing body of evidence that B cells possess the capability to modulate T cell activation and nonspecific immune responses [37], its role within the bone marrow microenvironment (BMM) has yet to be elucidated. Studies have shown that in AML there is a reduction of total B cells (CD19^+^ B cells) and an imbalance in the proportions of different B cell subpopulations: compared with healthy individuals, total B cells in the BM of relapsed or refractory AML patients are significantly reduced [38]; within one year after intensive cytotoxic chemotherapy, AML patients exhibit abnormal proportions of memory and transitional B cells in peripheral blood mononuclear cells (PBMCs) [39]; transcriptome sequencing analysis of BM samples from AML infants revealed that many significantly altered genes are associated with the development and differentiation of B cells [40]; the proportions of regulatory B cell (Breg) cells in PBMCs and bone marrow mononuclear cells (BMMCs) of AML patients are higher than healthy subjects, and overall survival of patients with a higher proportion of Breg cells are shorter [41]. These studies suggest that there is an imbalance of B cell subpopulations in PBMCs and BMMCs of AML patients. Alteration of proportions of different B cell subpopulations in BMM may exert a significant influence on the pathological process of AML, but the specific molecular mechanism has not been elucidated.

Although there are opinions that IDO1 is related to B cells dysfunction [42, 43], the effect of IDO1 on B cells remains to be explored. Herein, we aimed to clarify whether IDO1 is involved in the imbalance of B cell subpopulations in AML microenvironment, and whether IDO1 inhibitors are effective against AML. With clinical datasets and samples, the correlation between IDO1 and overall survival, immune cell proportions, especially B cell subpopulation proportions in AML patients were explored. The effect of IDO1 on B cells were studied in a co-culture system of healthy human PBMCs and AML cell line. Furthermore, the therapeutic efficacy of IDO1 inhibitors in C1498 AML bearing mice was examined. The present study represented a pioneering effort in demonstrating the impact of IDO1 on the imbalance of B cell subpopulations, which provided theoretical basis for immunotherapy of AML.

## Results

### IDO1 expression negatively correlated with survival and associated with distinct B Cell subpopulation proportions of AML patients

**Fig. 1:**
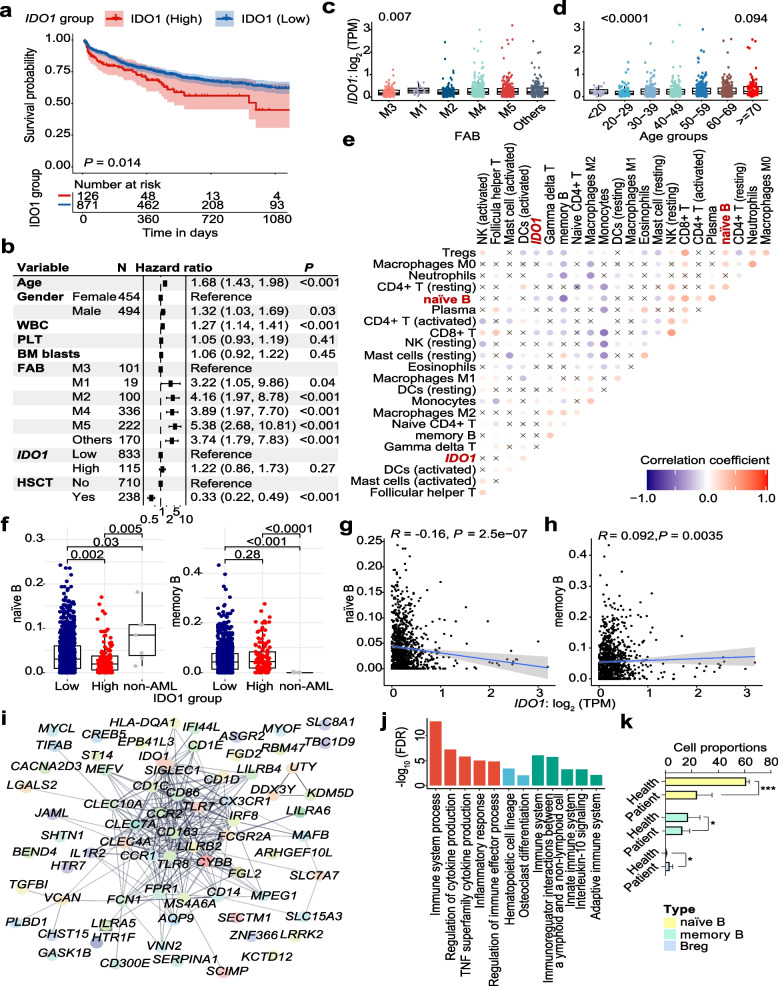
In AML patients, IDO1 expression negatively correlated to survival and the proportion of naïve B cells and positively correlated to the proportion of memory B cells. Clinical Data from Ruijin Hospital were analyzed. **a** Kaplan–Meier survival curves of OS of the AML patients according to *IDO1* mRNA expression level in bone marrow specimens. *IDO1*-low group (*n* = 871) and *IDO1*-high group (*n* = 126) were compared using the log-rank test. **b** Forest plot of multivariant analysis of basic clinical information and *IDO1* expression groups. **c** and **d** Boxplots of *IDO1* gene expression level in the FAB diagnosis and age groups of patients with AML. **e** The correlations between *IDO1* expression and relative proportions of 22 cell types in the BMM of AML patients using CIBERSORT method coupled with LM22. Fork marks indicate statistical significance below 0.05. **f** Box plots of naïve B cells and memory B cells fraction in *IDO1*-high AML group, IDO1-low AML group and non-AML group. **g** and **h** Scatter plots of naïve B cells(left), memory B cells fractions (right) and *IDO1* gene expression level (log_2_ (TPM + 1)). **i** STRING gene network of up-regulated genes in the high *IDO1* expression group. **j** Bar plot of enriched gene pathways using up-regulated genes in high *IDO1* expression group. **k** Flow cytometry analysis of the proportions of naïve B cells (CD19^+^IgD^+^CD27^−^), memory B cells (CD19^+^CD27^+^) and Breg cells (CD19^+^CD24^+^CD27^+^) in PBMCs from healthy individuals and AML patients. *n* = 3 per group. Statistical significance was determined by Student’s t-test and data were expressed as mean ± S.D., *p < 0.05, ***p < 0.001

To identify the prognostic value of IDO1 expression and the potential correlation of IDO1 expression with B cell subpopulation proportions in AML patients, we used the Ruijin Hospital dataset to compare the survival between *IDO1*-high group and *IDO1*-low group. Based on the gene expression level of *IDO1* in BM specimens (maximum rank statistic), AML patients from Ruijin Hospital were divided into low and high groups (Definition standards see Materials and Methods). Results suggested that AML patients with high *IDO1* expression had worse overall survival (*p* = 0.014) in AML (Fig. 1a). The *IDO1*-high group did not show independent prognosis significance (Fig. 1b) in AML patients. Subgroup analysis indicated that the gene expression level of *IDO1* was lower in AML-M3 subtype according to French American British (FAB) classification criteria (also known as acute promyelocytic leukemia) (Fig. 1c) and 20–29 age group (Fig. 1d), while the > = 70 years age group showed relatively higher gene expression of *IDO1* (Fig. 1d). The comparison between *IDO1-*low and *IDO1-*high groups indicated that high *IDO1* gene expression was associated with older age, lower BM blasts, HGB, and higher WBC level (Table S3). In addition, the positive rate of gene fusions was lower in *IDO1*-high group, in particular *CBFB::MYH11* and *RUNX1::RUNX1T1*, which are considered as relatively favorable genetic lesions of the ELN risk classification (Fig. S1). In contrast, the *TET2*, *FLT3*, *RUNX1*, *BCOR* and spliceosome mutations were more common in the *IDO1*-high group, which were commonly enriched in myelodysplastic syndrome (MDS)-transformed AML (Table S3).

CIBERSORTx-based deconvolution and the correlation analysis revealed the relationship between *IDO1* expression level and fractions of immune cells, including naïve B and memory B cells (Fig. 1e). The *IDO1*-high group showed significantly lower proportions of naïve B cells (Fig. 1f), plasma and CD8^+^ T cells (Fig. S2). On the contrary, the proportion of T cells suppressive monocytes was higher in the *IDO1*-high group (Fig. S2). Through Spearman correlation analysis, it was found that *IDO1* gene expression level is negatively correlated and positively correlated with the proportions of naïve B and memory B cells in BMM, respectively (Fig. 1g, h). Then, we identified 71 significantly up-regulated genes in the *IDO1*-high group in AML patients (Fig. 1i). Several immune-related gene pathways were found (Fig. 1j), including immune system process, regulation of cytokine production, and inflammatory response, etc. Gene expression and clinical data from the TCGA LAML patients (*n* = 151) [44] were used to successfully validate the prognosis significance of *IDO1* and the correlations between *IDO1* gene expression level and naïve and memory B cells proportions (Fig. S3).

To validate the abnormal proportions of B cell subpopulations in the immune microenvironment of AML patients, we employed flow cytometry to analyze B cells subpopulations proportions in PBMCs samples from 3 healthy individuals and 3 AML patients. The results revealed that the proportions of naïve B and memory B cells in PBMCs samples from AML patients were significantly lower than those in healthy individuals, while the proportion of Breg cells was significantly higher (Fig. 1k). In summary, these results suggested that *IDO1* gene expression inversely correlated with AML patient survival and associated with distinct B cell subpopulation proportions.

### IDO1 expression and activity correlated with B cell subpopulation proportions in BMMCs and PBMCs of AML patients

**Fig. 2:**
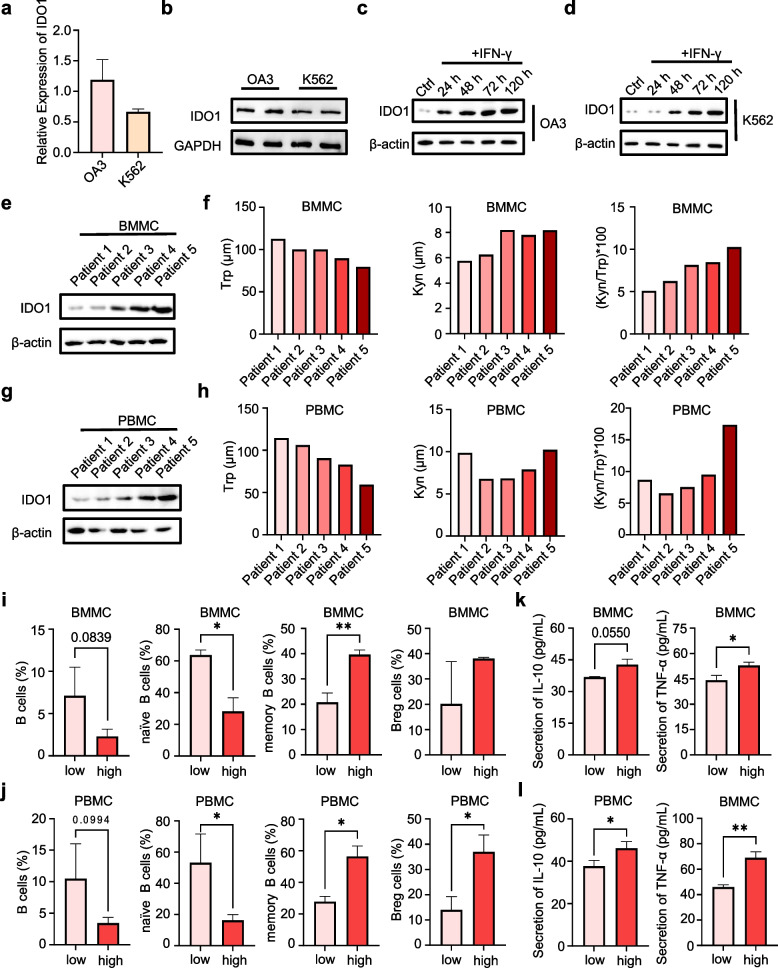
IDO1 constitutively expressed in AML cells and in BMMCs and PBMCs of AML patients, IDO1 expression correlated to B cell subpopulation proportions. **a** qPCR analysis of the mRNA expressions of IDO1 in K562 and OA3 cells, *n* = 3. **b** Western blot analysis of the protein expression of IDO1 in K562 and OA3 cells. **c**, **d** Western blot analysis of the expression of IDO1 in OA3 and K562 cells incubated with IFN-γ. **e**, **g** Western blot analysis of the protein expression of IDO1 in BMMCs and PBMCs from 5 patients with AML. **f**, **h** HPLC analysis of Trp and Kyn levels in medium of BMMCs and PBMCs from 5 patients with AML. **i**, **j** Flow cytometry analysis of the proportions of total B cells (CD19^+^), naïve B cells (CD19^+^IgD^+^CD27^−^), memory B cells (CD19^+^CD27^+^) and Breg cells (CD19^+^CD24^+^CD27^+^) in BMMCs and PBMC from 5 patients with AML. **k**, **l** ELISA analysis of IL-10 and TNF-α concentration in the medium of BMMCs and PBMCs from 5 patients with AML. Statistical significance was determined by Student’s t-test. Data were expressed as mean ± S.D., *p < 0.05, **p < 0.01

Although the mRNA expression of IDO1 has been recorded in the database, the protein expression of IDO1, particularly its pattern of expression in AML, remains inadequately characterized. Herein, two AML cell lines were chosen to investigate the expression dynamics of IDO1. As shown in Fig. 2a-b, both OA3 cells and K562 cells expressed IDO1, whereas IDO1 constitutive expression was higher in OA3 cells. IDO1 expression in both cell lines could be further induced by interferon-gamma (IFN-γ), and OA3 cells were more sensitive (Fig. 2c, d).

To investigate the expression pattern of IDO1 in AML and its impact on B cell subpopulation proportions, BMMCs and PBMCs samples from five patients with newly diagnosed AML were used. Based on the expression level of IDO1 in BMMCs and PBMCs, the patients were designated as Patient 1–5. Patient 1 had the lowest IDO1 expression, while Patient 5 had the highest IDO1 expression (Fig. 2e, g). The variation of KP activity was consistent with that of the IDO1 expression. Patient 5 had the lowest Trp level, the highest Kyn level and Kyn/Trp ratio in both BMMCs and PBMCs (Fig. 2f, h). We defined Patients 1 and 2 as the IDO1-low group, and Patients 3, 4, and 5 as the IDO1-high group. The IDO1-low group had the higher proportions of total B and naïve B cells, and the lower proportions of memory B and Breg cells in both BMMCs and PBMCs, while the data of the IDO1-high group were the opposite (Fig. 2i, j). ELISA detection of immunosuppressive cytokines also revealed that IDO1-high group had the higher levels of interleukin 10 (IL-10) and tumor necrosis factor alpha (TNF-α) in the BMMCs and PBMCs (Fig. 2k, l).

Collectively, these results depicted that higher IDO1 expression in BMMCs and PBMCs of AML patients was possibly associated with lower proportions of total B and naïve B cells, and higher proportions of memory B and Breg cells.

### IDO1 in AML cells affected the proportions and functions of B cell subpopulations in the Co-culture of PBMCs and AML cells

**Fig. 3:**
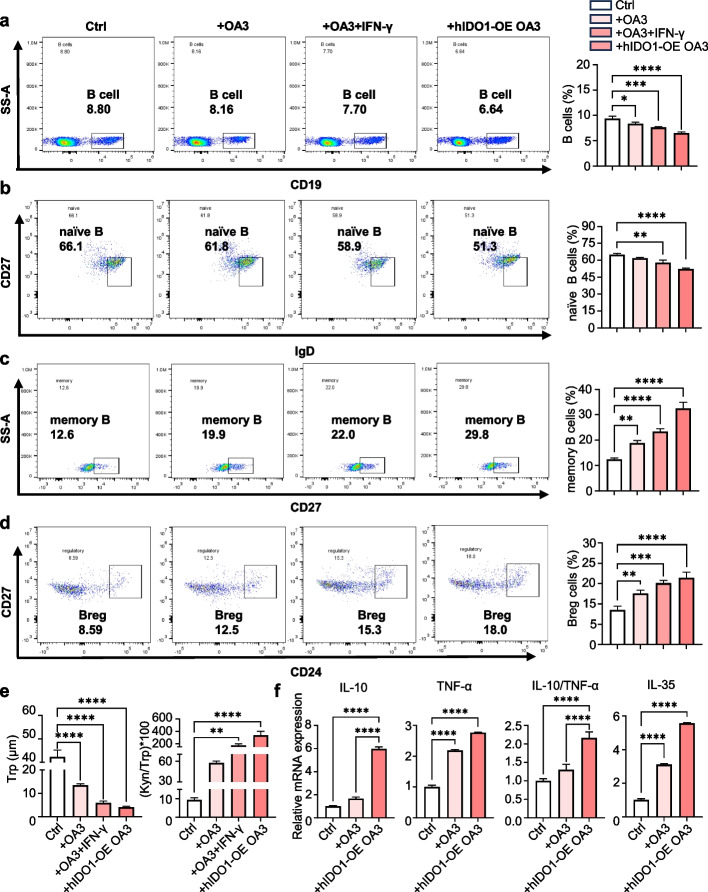
In co-culture of PBMCs and AML cells, high IDO1 expression reduced the proportions of total B and naïve B cells, increased the proportions of memory B and Breg cells. PBMCs were co-cultured with OA3 cells, IFN-γ treated OA3 cells, and hIDO1-OE OA3 cells, stimulated by IL-4 (10 ng/mL) and LPS (20 μg/mL) for 72 h. Ctrl represented PBMCs. **a**-**d** Flow cytometry analysis of the proportions of total B cells (CD19^+^), naïve B cells (CD19^+^IgD^+^CD27^−^), memory B cells (CD19^+^CD27^+^) and Breg cells (CD19^+^CD24^+^CD27.^+^). Representative histograms and quantification were shown. *n* = 3 per group. **e** HPLC analysis of Trp and Kyn levels in the supernatants. *n* = 3 per group. **f** qPCR analysis of relative expression of IL-10, TNF-α, IL-10/ TNF-α ratio and IL-35. *n* = 3 per group. Statistical significance was determined by one-way ANOVA followed by Dunnett’s post hoc test Data were presented as the mean ± SD. * *p* < 0.05, ** *p* < 0.01, *** *p* < 0.001

A co-culture of AML cells with healthy human PBMCs was established to simulate the tumour immune microenvironment and was used to test the effects of IDO1 in AML cells on B cell subpopulations including their proportions and functions. First, the B cell subpopulation proportions were compared among PBMCs alone, PBMCs co-cultured with OA3 cells, PBMCs co-cultured with IFN-γ treated OA3 cells, and PBMCs co-cultured with hIDO1-OE OA3 cells. As shown in Fig. 3a-d, both IFN-γ-induced and constitutive IDO1 expression in OA3 cells had significant effects on B cells and their subpopulations. The increase in IDO1 expression led to a decrease in the proportions of total B and naïve B cells, and an increase in the proportions of memory B and Breg cells. HPLC analysis also found that in the co-culture system containing IFN-γ treated OA3 cells and hIDO1-OE OA3 cells, the concentration of Trp was significantly reduced and KP activity was significantly increased (Fig. 3e). Recent studies have disclosed that the secretion of immunosuppressive cytokine IL-10 and IL-35 is contributed to the suppressive capability of Breg cells, and the ratio of IL-10 to TNF- α (IL-10/TNF-α) has been suggested serving as an anti-inflammatory index [45–47]. The levels of IL-35, IL-10, and TNF-α in PBMC were compared with different co-culture systems. As shown in Fig. 3f, the levels of TNF-α and IL-35 were higher in the co-culture of PBMCs and OA3 cells than in that of PBMCs alone, with the highest level in the co-culture of PBMCs and hIDO1-OE OA3 cells. Similarly, the level of IL-10 and IL-10/TNF-α were increased significantly in this co-cultured system.

**Fig. 4:**
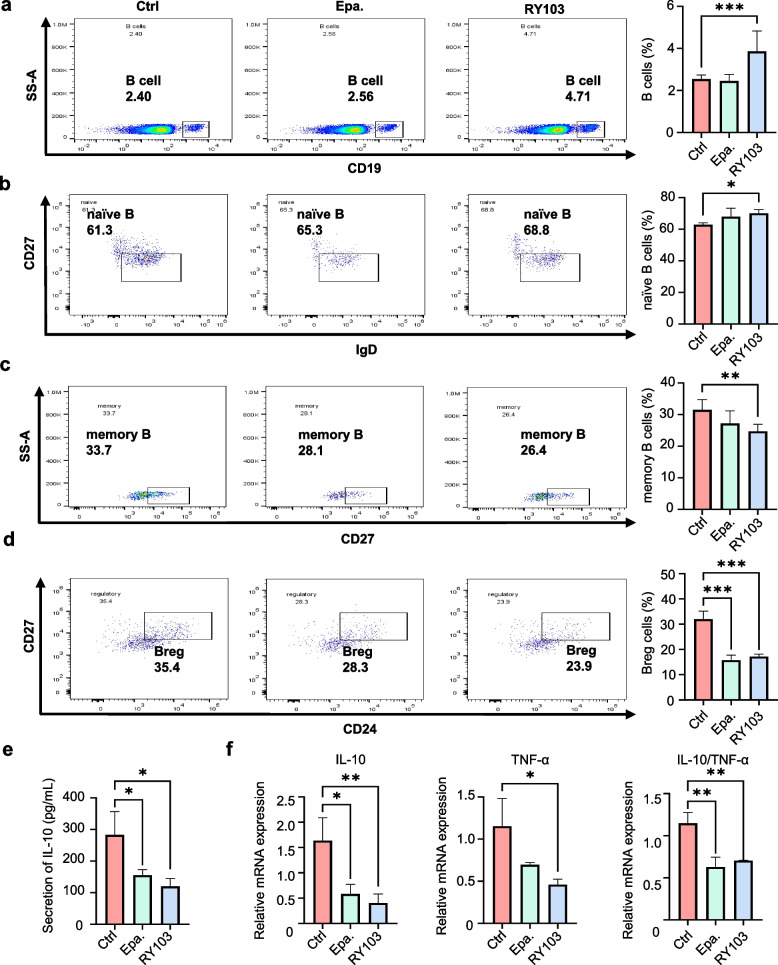
IDO1 inhibitors increased the proportions of total B and naïve B cells and decreased the proportions of memory B and Breg cells in the co-culture of PBMCs and AML cells. PBMCs were co-cultured with hIDO1-OE OA3 cells stimulated by IL-4 (10 ng/mL) and LPS (20 μg/mL), in the absence or presence of IDO1 inhibitor (2 μM) for 72 h. Ctrl represented the co-culture of PBMCs and hIDO1-OE OA3 cells, Epa. represented epacadostat. **a**-**d** Flow cytometry analysis of the proportions of total B cells (CD19^+^), naïve B cells (CD19^+^IgD^+^CD27^−^), memory B cells (CD19^+^CD27^+^), Breg cells (CD19^+^CD24^+^CD27^+^). Representative histograms and quantification of B cells were shown. *n* = 3 per group. **e** ELISA analysis of IL-10 concentration in the supernatants. *n* = 3 per group. **f** qPCR analysis of the levels of IL-10, TNF-α, and IL-10/TNF-α. *n* = 3 per group. Statistical significance was determined by one-way ANOVA followed by Dunnett’s post hoc test. Data were presented as the mean ± SD. * *p* < 0.05, ** *p* < 0.01, *** *p* < 0.001

With the co-culture of PBMCs and hIDO1-OE OA3 cells, the influence of IDO1 inhibitor on B cell subpopulations was investigated. First, the efficacy of epacadostat and RY103 on the blockade of KP were examined. The results indicated that either epacadostat or RY103 significantly suppressed the upregulated KP (decreasing Kyn levels and Kyn/Trp ratio) in hIDO1-OE OA3 cells (Fig. S5a), but they did not affect the KP activity in healthy PBMCs (Fig. S6a) and the co-culture system of PBMCs and OA3 cells (Fig. S7e). In addition, neither epacadostat nor RY103 appeared to affect the viabilities of hIDO1-OE OA3 cells and healthy PBMCs (Fig. S5b, S6b). Then, the effects of epacadostat and RY103 on the B cells and its subpopulations in the co-culture were investigated. As shown in Fig. 4a-d, the supplement of epacadostat and RY103 increased the proportions of total B and naïve B cells while decreased the proportions of memory B and Breg cells. On the contrary, IDO1 inhibitors did not alter the proportions of B cell subpopulations in PBMCs alone (Fig. S6c-f) and the co-culture of PBMCs and OA3 cells (Fig. S7a-d), which confirmed the important immunoregulatory role of IDO1 on B cells. The levels of IL-10 and TNF-α as well as IL-10/TNF- α in the co-culture of PBMCs with hIDO1-OE OA3 cells were reduced by the treatment of epacadostat or RY103 (Fig. 4e, f).

These results indicated that high expression of IDO1 in AML cells reduced the proportions of total B and naïve B cells, increased the proportions of memory B and Breg cells, and suppressed the expression of cytokines in the co-culture of PBMCs and AML cells.

### IDO1 inhibitors exhibited anti-leukemic effect in AML mice

**Fig. 5:**
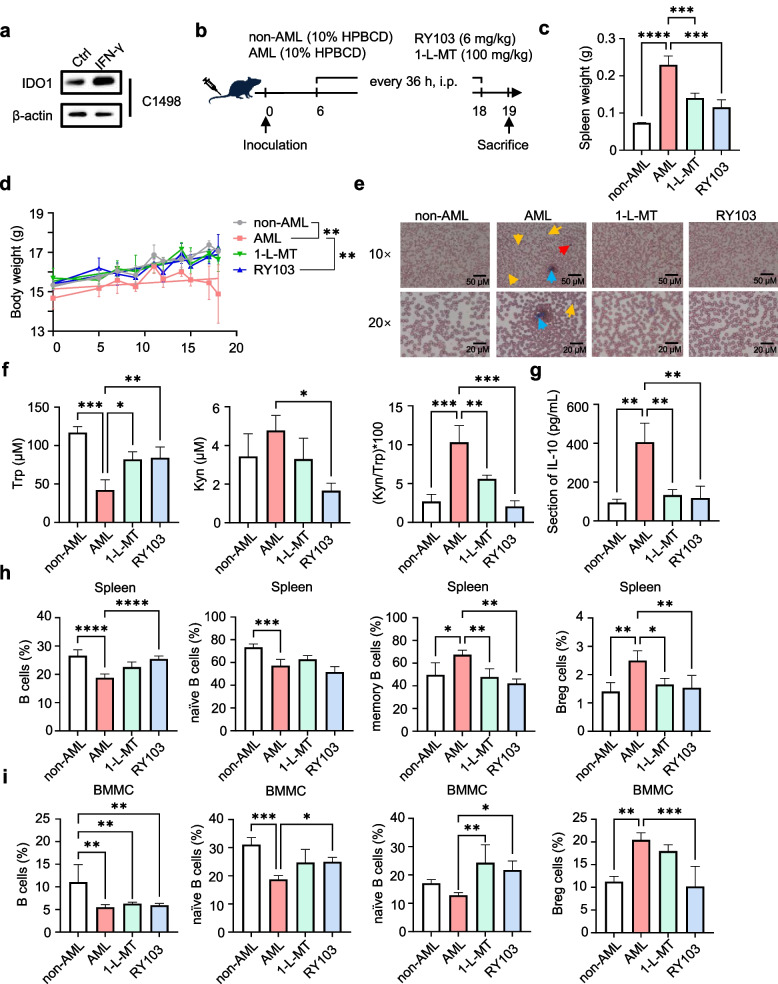
IDO1 inhibitors inhibited tumor growth and ameliorated the abnormal proportions of B cell subpopulations in C1498 AML-bearing mice. **a** Western blot analysis of the protein expressions of IDO1 in C1498 cells with or without IFN-γ (100 ng/mL) for 48 h. **b** Schematic representation illustrating the tumor inoculation in C57BL/6 female mice with 1 × 10^6^ C1498 cells per mouse. The construction of mouse models and the designation of the different treatments were described in the Materials and Methods section. **c** Spleens isolated from mice were weighted, *n* = 3–5 per group. **d** Body weight was recorded every 36 h and statistical analysis was shown, *n* = 3–5 per group. **e** Representative images of morphology of peripheral blood cells after Wright-Giemsa staining from different groups, *n* = 3–5 per group. The red arrow represents red blood cells, the yellow arrow represents AML blast cells, and the blue arrow represents neutrophils. Three photos were taken from the peripheral blood smear of each mouse, The magnification of the microscope is 10 and 20 times, and the scales are 50 and 20 µm, respectively. **f** HPLC analysis of serum Trp and Kyn levels, and Kyn/Trp ratio was calculated, *n* = 3–5 per group. **g** ELISA analysis of serum IL-10 levels from different groups, *n* = 3–5 per group. **h**-**i** Flow cytometry analysis of the proportions of total B cells (CD19^+^), naïve B cells (CD19^+^IgD^+^CD27^−^), memory B cells (CD19^+^CD27^+^) and Breg cells (CD19^+^CD24^+^CD27.^+^) in spleen (**h**) and bone marrow (**i**) from different groups, *n* = 3–5 per group. Statistical significance was determined by one-way ANOVA followed by Dunnett’s post hoc test. Data were presented as the mean ± SD. * *p* < 0.05, ** *p* < 0.01, *** *p* < 0.001

To validate the regulatory effect of IDO1 on the proportion and function of B cells in AML in vivo, we conducted experiments using C1498 cells in AML mouse model. C1498 cells are murine AML cells that can express IDO1 (Fig. 5a). We carried out the in vivo anti-leukemic assay of IDO1 inhibitors (1-L-MT and RY103) in C1498 AML-bearing mice. (Fig. 5b). At the end of two-week treatment, IDO1 inhibitors restored the body weight loss caused by AML to a similar level of non-AML mice (Fig. 5d). Similarly, IDO1 inhibitors reduced spleen weight (Fig. 5c). As a typical and diagnostic manifestation, the accumulation of leukemic blast cells in peripheral blood along with the decreased red blood cell count and mature neutrophil count occur in AML. As shown in Fig. 5e, the treatment with IDO1 inhibitors restored the elevated levels of leukemia blast cells and lower levels of red blood cells and mature neutrophils in AML model mice to the levels similar to non-AML. Next, the effects of RY103 and 1-L-MT on KP blockade in serum were assessed. As shown in Fig. 5f, compared to non-AML mice, serum Trp concentration was significantly reduced and the Kyn/Trp ratio was significantly elevated in AML mice. Meanwhile, serum Trp concentration in 1-L-MT group and RY103 group were higher and the Kyn/Trp ratio was lower compared to those in AML group. Taken together, our results indicated that IDO1 inhibitors suppressed the tumor growth in C1498 AML-bearing mice.

Furthermore, to determine whether the suppression of tumor growth in C1498 AML-bearing mice caused by IDO1 inhibition was related to changes in the proportions and functions of B cell subpopulations, we examined the proportions of B cell subpopulations in spleen and BM and the concentration of IL-10 in serum. Our results showed that in the spleen, the proportions of total B and naïve B cells in AML mice decreased and the proportions of memory B and Breg cells increased significantly compared to non-AML mice. After treatment with IDO1 inhibitors, the abnormal proportions of total B, memory B and Breg cells were recovered, and the effect of RY103 was better between the two (Fig. 5h). Similar results were observed in the BM as in the spleen, and treatment with IDO1 inhibitors significantly recovered the abnormal proportions of naïve B, memory B, and Breg cells (Fig. 5i). At the same time, we found that the proportion of CD8^+^ T cells in AML-bearing mice was significantly reduced. However, IDO1 inhibitors were unable to recover it, further confirming that IDO1 inhibitors reversed the immunosuppressive state of mice by ameliorating the abnormal proportions of B cell subpopulations (Fig. S8). Besides, the concentration of IL-10 in the serum of AML mice was significantly higher than that of non-AML mice (Fig. 5g). It has also been shown that this increase was reversed by IDO1 inhibitions.

In summary, our results indicated that IDO1 played a crucial role in AML progression, affecting B cell subpopulations proportions and cytokine levels, ultimately altering the immune status. IDO1 inhibitors showed promising anti-leukemic effects.

### IDO1 potentially affected the proportions and functions of B cell subpopulations in AML through PI3K-AKT signaling pathway

**Fig. 6:**
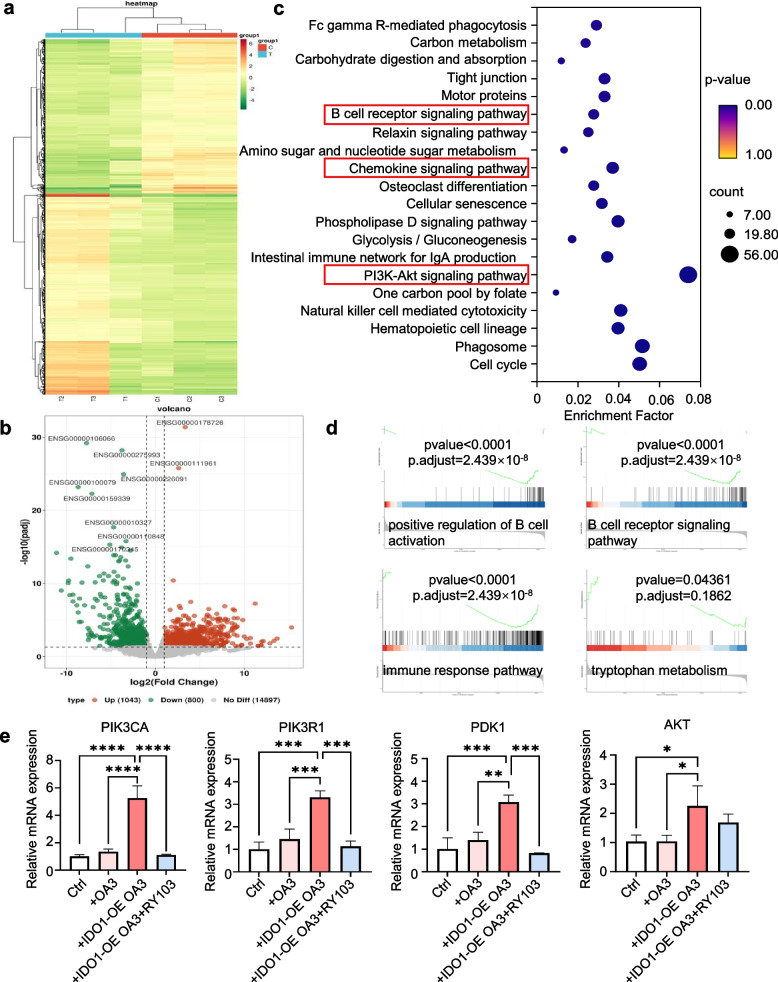
IDO1 activated PI3K-AKT signaling pathway to affect the proportions and functions of B cell subpopulations in the co-culture of PBMCs and hIDO1-OE OA3 cells. RNA sequencing analysis was performed on PBMCs and PBMCs co-cultured with hIDO1-OE OA3 cells. **a** Differential expression gene clustering analysis heatmap. **b** Differentially expressed genes were presented as a volcano plot. The software for differential expression analysis is DESeq2, with a screening threshold of padj < 0.05 and |log2FoldChange|> 1. **c** KEGG Pathway enrichment analysis of differentially expressed genes. **d** GSEA Pathway enrichment analysis results. Perform GSEA enrichment analysis on GO and PATHWAY content using cluster Profile, respectively. **e** qPCR analysis of the levels of *PIK3CA, PIK3R1, PDK1* and *AKT* in the PBMCs, PBMCs co-cultured with OA3 and hIDO1-OE OA3 cells, PBMCs co-cultured with RY103 treated hIDO1-OE OA3 cells. Ctrl represented PBMCs. *n* = 3 per group. Statistical significance was determined by one-way ANOVA followed by Dunnett’s post hoc test. Data were presented as the mean ± SD. * *p* < 0.05, ** *p* < 0.01, *** *p* < 0.001

Given that we had found in the above-mentioned studies that IDO1 in AML cells can affect the proportions and functions of B cell subpopulations in AML, we attempted to elucidate the specific mechanisms involved. RNA-sequencing analysis was performed and compared on healthy PBMCs and the co-culture of PBMCs with hIDO1-OE OA3 cells. 1843 differentially expressed genes were detected, of which 1043 were upregulated and 800 were downregulated (Fig. 6a-b). Downregulated genes were predominantly clustered in genes related to lymphocytes, including *SASH1, STAB1, CD69,* and *FOS*. The Kyoto Encyclopedia of Genes and Genomes (KEGG) pathway enrichment analysis demonstrated that the genes with the most significant differential expression were annotated to the PI3K-AKT pathway. (Fig. 6c). Class IA PI3K activates the expression of B cell transcription factor Pax5, making it necessary for B cell differentiation [48], and in a recently published work, the PI3K-AKT signaling pathway has been also found in age-related genes of AML patients, which involves inflammation and inhibitory immune microenvironment [49]. In addition to the PI3K pathway, our results also identified several pathways related to immune responses, including the B cell receptor (BCR) signaling pathway and cytokine signaling pathway, as well as other pathways related to cell proliferation and differentiation. We validated through qPCR experiments that high IDO1 expression increased mRNA expression of PIK3CA, PIK3R1 subunits of PI3K kinase and Akt, while the addition of IDO1 inhibitors eliminated this phenomenon in the co-culture of PBMCs and hIDO1-OE OA3 cells (Fig. 6e).

GSEA enrichment analysis was performed on KEGG Pathway, and it was found that high expression of IDO1 was negatively correlated with positive regulation of B cell activation pathway, BCR signaling pathway, and immune response pathway (Fig. 6d). GSEA enrichment was performed on Gene Ontology (GO) terms, and the results also showed significant enrichment of pathways related to tryptophan metabolism and B cell receptor signaling pathways.

## Discussion

B cell subpopulations imbalance has been observed in PBMCs and BMMCs of AML patients [9, 50], which may contribute to the pathological progression and immune evasion of AML, but the specific molecular mechanism behind has not been elucidated. We speculate that the imbalance of B cell subpopulations is potentially related to IDO1 that has been suggested to be involved in AML immune evasion.

In the current research, we analyzed the correlation between IDO1 expression and the proportions of B cell subpopulations using AML patient samples at both bioinformatics and clinical data levels. We found that IDO1 expression and activity were negatively associated with the proportions of total B and naïve cells, and positively correlated with the proportions of memory B and Breg cells (Figs. 1, 2). Existing studies only focus on the relationship between IDO1 and AML prognosis, as well as that between B cells subpopulations imbalance and AML, but not the relationship between IDO1 and the imbalance of B cell subpopulations. We demonstrated for the first time that IDO1 expression corelated with the proportions of B cell subpopulations.

Subsequently, we also validated the relationship between IDO1 and B cells in vitro. We used co-culture of healthy human PBMCs and AML cells to better simulate the tumor microenvironment. The results showed that high IDO1 expression could reduce the proportions of total B and naïve B cells, increase the proportions of memory B and Breg cells, and the levels of immunosuppressive cytokines (Fig. 3). The supplement of IDO1 inhibitors epacadostat and RY103 could restore these abnormal proportions of B cells subpopulations (Fig. 4). Co-culture of AML cells and immune cells such as macrophages and T cells have been usually utilized [51]. In this study, we first used co-culture models to study the proportions of B cell subpopulations. However, this co-culture model still had some limitations. Since the two types of cells were physically in contact with each other, it was difficult to separate each types of cells for further downstream assays [51].

We further investigated the signaling pathways that may be involved in the regulation of IDO1 on proportions of B cell subpopulations through RNA-seq, and found that the PI3K-AKT pathway had the most significant correlation with IDO1 (Fig. 6). It has been reported that the PI3K-AKT pathway has a crucial role in the progression of AML, promoting the proliferation and spread of AML blast cells through different mechanisms such as inhibiting cell apoptosis, enhancing the migration and invasion ability of AML blast cells [52, 53]. It has also been shown that IDO1 binds to the PI3K p85 subunit via a YxxM motif [54]. Our results connected IDO1 with the PI3K pathway, expanding the knowledge on regulatory role of IDO1 in AML.

IDO inhibitor has been a research and development pipeline of well-known pharmaceutical companies in the past few years. Approximately 100 clinical trials of at least 8 small molecule IDO1 inhibitors aiming to characterize the safety and efficacy profile have been initiated and the majority of them focused on solid tumors [11]. It should be emphasized that very few research has focused on AML. In our study, the anti-tumor activity of IDO1 inhibitors RY103 and 1-L-MT were tested in C1498 AML bearing mice. The results showed that RY103 had better ability to block KP and better anti-tumor efficacy than 1-L-MT in AML mice. RY103 reversed the immunosuppressive state of AML mice by ameliorating the abnormal proportions of B cell subpopulations (Fig. 5). Previous reports have shown that various lymphocytes including NK cells and T cells are inhibited in AML’s BMM [55–57]. Our study revealed for the first time the effect of IDO1 on the proportions and functions of B cell subpopulations, providing a theoretical basis for IDO1 inhibitor therapy for AML.

Our inability to utilize more AML patient samples and AML cell lines indicates that our research results are not perfect. Besides, our future study may explore the mechanism by which IDO1 regulates B cell differentiation via PI3K-AKT pathway.

**Fig. 7:**
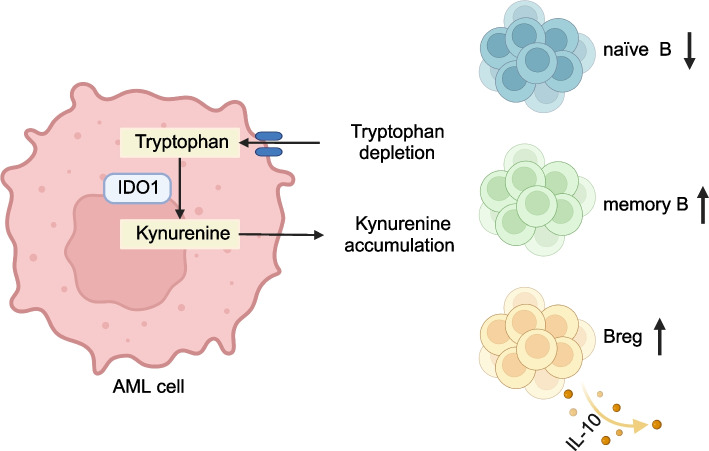
The schematic demonstration of IDO1 expression contributes to the malignancy of acute myeloid leukemia by affecting the proportions of B cell subpopulations. IDO1 in AML blast cells depletes Trp and accumulates Kyn in the TME, thereby altering the proportions and functions of B cell subpopulations, specifically manifested as a decrease in the proportion of naïve B cells, an increase in the proportions of memory B and Breg cells and the expression of immunosuppressive cytokines

In summary, our study revealed the impact of IDO1 on both the proportions of B cell subpopulations and immunosuppressive cytokines within BMM and therefore provided new evidence for the contribution of IDO1 in immune evasion, extending beyond its well-recognized impact on the T cells in AML (Fig. 7). Our study elucidated the potential therapeutic potential of IDO1 inhibitor as a valuable treatment of AML, which warranted the proof from clinical study.

## Materials and methods

### Bioinformatic analysis

Previously published 1,007 adults AML from Ruijin Hospital Affiliated to Shanghai Jiao Tong University School of Medicine [49] were included in this study. Available prognosis data was obtained in more than 99% (*n* = 997) patients. To validate the conclusions, we also reproduced the core results based on the public data from TCGA LAML patients (*n* = 151). The gene expression level was normalized using TPM (transcripts per million) measures. Using the Surv-Cutpoint function in the survminer package of R, which repeatedly tested all potential cut points to establish the maximum rank statistic, the patients were divided into two groups: the high IDO1 expression group (IDO1-high) and the low IDO1 expression group (IDO1-low). Immune cell deconvolution was performed using the CIBERSORTx software [58]. Spearman correlation analysis was used to infer potential interactions between gene-immunocyte pairs. Differentially expressed genes were calculated using DESeq2 variance stabilizing transformation (VST) normalization and the Limma package. The gene sets enrichment analysis was performed using the clusterProfiler [59] and Broad GSEA programs (adjusted *p* < 0.05 and log2 (fold change) > 1).

### Human samples

BM and PB samples were obtained from 5 patients with non-promyelocytic AML at Ruijin Hospital Affiliated to Shanghai Jiao Tong University School of Medicine and used to isolate BMMCs and PBMCs. PBMCs of healthy individuals were purchased from Milestone Biotechnologies company. The baseline and disease characteristics information was provided in Table. S1 (for Supplementary information).

### Cell culture

OCI-AML3 (human AML cell line, OA3) and K562 (human chronic myeloid leukemia cell line) were procured from Shanghai Institute of Hematology. C1498 (mouse AML cell line) was purchased from Haixing Biosciences Co., Ltd. OA3, K562 and C1498 cells were cultured in RPMI 1640 (Gibco, Cat# 31800022) supplemented with 10% FBS, 100 U/mL of penicillin (Aladdin, Cat# S432673) and 100 μg/mL of streptomycin (Aladdin, Cat# S432673). Cells were grown at 5% CO2 in a humidified incubator at 37℃.

### In vitro PBMCs co-culture with AML cells

Healthy human PBMCs were co-cultured with OA3 cells stably over-expressing IDO1 (hIDO1-OE OA3) or WT OA3 cells to simulate the tumor immune microenvironment, and the ratio of PBMCs to AML cells in the co-culture system was 5:1, and the system was stimulated by recombinant human interleukin (IL)−4 Protein (Yeasen, Cat# 90105ES08) at 10 ng/ml and lipopolysaccharide (LPS) (Sigma, Cat# L2880) at 20 μg/ml for 72 h.

### Reagents

HPβCD ((2-hydroxypropyl)-β-cyclodextrin, Aladdin, Cat# H108813), 1-L-MT (Sigma-Aldrich, Cat# 447,439), epacadostat (INCB024360, Selleck, Cat# S7910), murine Interferon-γ (IFN-γ, Beyotime, Cat# P6137), human IFN-γ (Yeasen, Cat# 91211ES10) were used in this study. RY103 was designed and synthesized by our lab.

### Cell treatment conditions

OA3 and K562 cells: incubated with 100 ng/mL of human IFN-γ for 24/48/72/120 h.

C1498 cell: incubated with 100 ng/mL of murine IFN-γ for 48 h.

hIDO1-OE OA3, PBMCs co-culture with hIDO1-OE OA3 cells: RY103 (incubated with 2 μM of RY103 for 72 h); epacadostat (incubated with 2 μM of epacadostat for 72 h).

### Animal model and treatment

Female, 4 to 5-week-old, C57BL/6 mice were purchased from Shanghai ShengChang.

The mice were divided into non-AML and AML groups. To construct AML mice, C1498 cells (1 × 10^6^ cells /mouse) were intravenously injected. Beginning on day 6 after the implantation, mice were randomized and given the treatments intraperitoneally (i.p.) on the same day. Treatments were listed as follows: non-AML and AML mice received 10% HPβCD. Mice RY103 and 1-L-MT groups received 6 mg/kg of RY103 in 10% HPβCD and 100 mg/kg of 1-L-MT in 10% HPβCD, respectively every 36 h. Once the two-week treatment was completed, the mice were sacrificed. The spleen was isolated and weighted, and the BM was collected. B cells in spleen and BM were detected by flow cytometry.

### High-performance liquid chromatography (HPLC) analysis of Trp and Kyn

The cell culture supernatant and mice serum were treated with 5% perchloric acid precipitation protein method. After centrifugation, the supernatant was taken and the content of Trp and Kyn was detected by HPLC based on the retention time and the UV absorption (280 nm for Trp, 360 nm for Kyn) [25]. (Kyn/Trp) × 100 reflects the activity of IDO1.

### RT-PCR and quantitative real-time PCR (qPCR)

qPCR was performed by using the SYBR Green PCR Master Mix kit (ABclonal, Cat# RK21203) for the detection of *IDO1, IL-10, TNF-α, IL-35, PIK3CA, PIK3R1, PDK1, AKT, β-actin* mRNA expression. The primers used for the qPCR are shown in Table S2.

### Western blot (WB)

Protein samples obtained from AML cells were transferred onto PVDF membranes after separated by SDS-PAGE. The following primary antibodies against anti-IDO1 (1:3000, Proteintech, Cat# 66,528–1-Ig), anti-GAPDH (1:10,000, Proteintech, Cat# 60,004–1-Ig) and anti-β-actin (1:5000, ABclonal, Cat# AC004) antibodies were used. The proteins were treated with an HRP-conjugated secondary antibody and were visualized using ECL reagents (Tanon, Cat# 1,805,001).

### Enzyme-linked immunosorbent assay (ELISA)

The concentrations of IL-10 in the medium and serum were measured by ELISA kit (HUABio, Cat# EM0005 and EH0009). All procedures were performed following the product manual.

### Cell counting kit-8 (CCK-8) assay

After treating the cells according to the above “Materials and Methods 4.6”, the cell culture medium was replaced with fresh culture medium containing 10% CCK-8 solution. After culturing the cells for 4 h, the absorbance at a wavelength of 450 nm was measured using a microplate reader.

### Flow cytometry analysis

Cells from co-culture system, BMs and spleens of mice were labeled with the following fluorescence-conjugated antibodies purchased from Biolegend: PerCP/Cyanine5.5 anti-human CD19 (Cat# 302,230), FITC anti-human IgD (Cat# 348,206), PE anti-human CD24 (Cat#311,106), PE/Cyanine7 anti-human CD27 (Cat#302,838), PerCP/Cyanine5.5 anti-mouse CD19 (Cat#152,405), FITC anti-mouse IgD (Cat# 405,703), PE anti-mouse CD24 (Cat#101,807), PE/Cyanine7 anti-mouse CD27 (Cat#124,215), FITC anti-mouse CD45 (Cat# 103,108), APC anti-mouse CD3 (Cat#100,235), PE anti-mouse CD8α (Cat#100,707). Zombie NIR Fixable Viability Kit (Biolegend, Cat# 423,105) was used to label viable cells. Cells with different labels were detected using the Beckman Coulter’s Gallios flow cytometer, and the data were analyzed using FlowJo software. Flow cytometry gating strategies were shown in Fig. S4 (for Supplementary information).

### Wright–Giemsa staining

The slides were soaked in 10% hydrochloric acid for 24 h in advance and washed thoroughly to dry. 10 μL of fresh mouse blood was evenly spread on a glass slide and placed in a 37 °C incubator to keep warm and dry. The rapid Wright-Giemsa staining solution (Yisheng, Cat# 60529ES01) was used for post-staining observation. All procedures were performed following the product manual.

### RNA sequencing

RNA from the co-culture of PBMCs and IDO1-OE OA3 cells (experimental group) and PBMCs alone (control group) were extracted (*n* = 3 in each group). Library construction and sequencing were performed on an Illumina platform by Wuhan Benagen Technology. The raw data were first processed with FastQC (v0.11.9, default) to filter out adapters and low-quality sequences. Then, clean reads were mapped to the human genome using Star (v2.7.9a, default). Gene expression level for each sample were calculated using RSEM (v1.3.3, default), and expressed as fragments per kilobase of transcript per million fragments mapped. The gene expression levels in stressed samples were analyzied againt those in control samples to identify the differentially expressed genes (DEGs) using DESeq2 (v1.34.0, default). The DEGs were identified as previously described, using the parameters: Foldchange ≥ 2.00 and Probability ≥ 0.8 with a significant false discovery rate-adjusted *P* value < 0.05 based on the three biological replicates. GO and KEGG enrichment analyses for the DEGs were conducted using cluster Profiler version 3.8.

### Statistical analysis

All experiments were repeated at least three times. Prism 10 software (GraphPad Software) was used to create graphs and perform statistical analyses. Data were expressed as the means ± SD. One-way analysis of variance (ANOVA) was used to compare several treatment groups with control groups. Student’s t-test was used to determine the difference between two groups. Significance values were set at *p < 0.05, **p < 0.01, ***p < 0.001 and ****p < 0.0001.

## Supplementary Information

Supplementary Material 1: Fig. S1. Differential gene comparison between *IDO1*-low and high groups of AML patients from Ruijin hospital. Each row represents a clinical or genomic characteristic. Each column represents one patient. The patients are divided into left (low) and right (high) panels according to the gene expression level of IDO1. The cutoff of low and high is determined by the prognosis significance. The right percentages and bar plots show the overall mutation rate and the number of mutations (genes or pathways). Fig. S2. Other significant difference in immune fractions between *IDO1*-low and high groups of AML patients from Ruijin hospital. The statistical significance is calculated based on the Wilcoxon Rank Sum Test. Fig. S3. Correlations between *IDO1* gene expression and clinical prognosis, and between *IDO1* gene expression and proportions of naïve/memory B cells in the TCGA LAML cohort. (a) Kaplan-Meier survival curves of OS of the AML patients according to *IDO1* mRNA expression level in bone marrow specimens. IDO1-low group (*n* = 71) and IDO1-high group (*n* = 80) were compared using the log-rank test; (b) Forest plot of multivariant analysis of basic clinical information and *IDO1* expression groups; (c) The correlations between *IDO1* gene expression and relative proportions of 22 cell types in the BMM of AML patients from the TCGA LAML database using CIBERSORT method coupled with LM22. Fork marks indicate statistical significance below 0.05; (d) Scatter plots of the proportions of naïve (left), memory B cells (right) and *IDO1* gene expression level (log2 (TPM+1)). Fig. S4. Flow cytometry gating strategy. (a) FCM gating strategy for detection of total B cells (CD19^+^) cells, naïve B cells (CD19^+^IgD^+^CD27^-^), memory B cells (CD19^+^CD27^+^), Breg cells (CD19^+^CD27^+^CD24^+^). (b) FCM gating strategy for detection of CD8^+^ T cells (CD45^+^CD3^+^CD8^+^). All gates were set using fluorescence-minus-one (FMO) controls. Fig. S5. IDO1 inhibitors significantly inhibited the upregulated KP without cytotoxicity in hIDO1-OE OA3 cells. (a) HPLC analysis of Trp and Kyn levels in medium from OA3 cells (Ctrl), hIDO1-OE OA3 cells and IDO1 inhibitor (2 μM) treated hIDO1-OE OA3 cells. (b) CCK-8 analysis of cytotoxicity of IDO1 inhibitor on hIDO1-OE OA3 cells (Ctrl). Epa. represented epacadostat. Statistical significance was determined by one-way ANOVA followed by Dunnett’s post hoc test. Data were presented as the mean ± SD. * *p*< 0.05, ** *p* < 0.01, *** *p* < 0.001. Fig. S6. IDO1 inhibitor did not affect the basal KP and the proportions of B cell subpopulations without cytotoxicity in healthy human PBMCs. PBMCs were treated with IDO1 inhibitor (2 μM unless stated otherwise) stimulated by IL-4 (10 ng/mL) and LPS (20 μg/mL) for 72 h, Epa. represented epacadostat. (a) HPLC analysis of Trp and Kyn levels in medium. (b) CCK-8 analysis of the viability of PBMCs. (c-f) Flow cytometry analysis of the proportions of total B cells (CD19^+^), naïve B cells (CD19^+^IgD^+^CD27^-^), memory B cells (CD19^+^CD27^+^) and Breg cells (CD19^+^CD24^+^CD27^+^). Fig. S7. RY103 only changed the proportions of B cell subpopulations and KP activity in the co-culture of PBMCs and hIDO1-OE OA3 cells. PBMCs were co-cultured with OA3 cells or hIDO1-OE OA3 cells stimulated by IL-4 (10 ng/mL) and LPS (20 μg/mL), in the absence or presence of RY103 (2 μM) for 72 h. (a-d) Flow cytometry analysis of the proportions of total B cells (CD19^+^), naïve B cells (CD19^+^IgD^+^CD27^-^), memory B cells (CD19^+^CD27^+^) and Breg cells (CD19^+^CD24^+^CD27^+^). Representative histograms and quantification were shown. *n*=3 per group. (e) HPLC analysis of Trp and Kyn levels in medium. Statistical significance was determined by one-way ANOVA followed by Dunnett’s post hoc test for comparison. Data were presented as the mean± SD. * *p* < 0.05, ** *p* < 0.01, *** *p* < 0.001. Fig. S8. IDO1 inhibitor did not alter the proportion of CD8^+^ T cells in the spleen and bone marrow of AML mice. The construction of mouse models and the assignment of the different treatments were described in the Materials and Methods section. (a-b) Flow cytometry analysis of the proportion of CD8^+^T cells (CD45^+^CD8^+^) in spleen and BMMCs, *n*=5 per group. Representative histograms and quantification were shown. Statistical significance was determined by one-way ANOVA followed by Dunnett’s post hoc test. Data were presented as the mean ± SD. * *p* < 0.05, ** *p*< 0.01, *** *p* < 0.001. Table S1. Clinical characteristics of 5 AML patients from Ruijin Hospital included in this study. Table S2. PCR Primers for quantification. Table S3. Clinical and molecular features in IDO1-low and high groups of AML patients from Ruijin hospital.

Supplementary Material 2.

## Data Availability

Anonymized RNA sequencing data of Ruijin AML database is available in The Genome Sequence Archive for Human (GSA-Human, https://ngdc.cncb.ac.cn/gsa-human) referring to HRA002693 and HRA006117. TCGA-LAML data analyzed in this study is publicly available on the national cancer institute (NCI) genomic data commons (GDC) portal (https://portal.gdc.cancer.gov/). Experimental data generated or analyzed in this study are included in this published article and its supplementary information files. Additional material used and/or analyzed during the current study are available from the corresponding author on reasonable request.

## Abbreviations

AML: Acute myeloid leukemia
ANOVA: One-way analysis of variance
ARG2: Arginase-2
BM: Bone marrow
BMM: Bone marrow microenvironment
BMMCs: Bone marrow mononuclear cells
Bregs: Regulatory B cells
CAR: Chimeric antigen receptor
CCK-8: Cell counting kit-8
DCs: Dendritic cells
ELISA: Enzyme-linked immunosorbent assay
Epa: Epacadostat
FBS: Fetal bovine serum
FCM: Flow cytometry
HPβCD: 2-Hydroxypropyl-β-cyclodextrin
HPLC: High-performance liquid chromatography
IDO1: Indoleamine 2,3-dioxygenase 1
IDO1-OE OA3: IDO1 over-expressing OA3 cells
IFN-γ: Recombinant murine/human Interferon-γ
IL-4: Recombinant Human interleukin-4
i.p.: Intraperitoneally
Kyn(s): Kynurenines
LPS: Lipopolysaccharide
MDCSs: Myeloid-derived suppressor cells
MDS: Myelodysplastic syndrome
NK cells: Natural killer cells
OA3: OCI-AML3 cells
OS: Overall survival
PB: Peripheral blood
PBMCs: Peripheral blood mononuclear cells
PBS: Phosphate-buffered saline
qPCR: Quantitative real-time PCR
RPMI 1640: Roswell Park Memorial Institute
TAMs: Tumor-associated macrophages
TCGA: The Cancer Genome Atlas
TME: Tumor microenvironment
Tregs: Regulatory T cells
Trp: Tryptophan
WB: Western blot
WT: Wild type
