# Supplementary material for: Indoleamine 2,3-dioxygenase 1 alters the proportions of B cell subpopulations in the microenvironment of acute myeloid leukemia
Source: Mol Biomed. 2025 Apr 16;6:23. doi: 10.1186/s43556-025-00262-x (PMC12000501; doi:10.1186/s43556-025-00262-x)
Supplement: Supplementary file 1 — Supplementary Material 1: Fig. S1. Differential gene comparison between IDO1-low and high groups of AML patients from Ruijin hospital. Each row represents a clinical or genomic characteristic. Each column represents one patient. The patients are divided into left (low) and right (high) panels according to the gene expression level of IDO1. The cutoff of low and high is determined by the prognosis significance. The right percentages and bar plots show the overall mutation rate and the number of mutations (genes or pathways). Fig. S2. Other significant difference in immune fractions between IDO1-low and high groups of AML patients from Ruijin hospital. The statistical significance is calculated based on the Wilcoxon Rank Sum Test. Fig. S3. Correlations between IDO1 gene expression and clinical prognosis, and between IDO1 gene expression and proportions of naïve/memory B cells in the TCGA LAML cohort. (a) Kaplan-Meier survival curves of OS of the AML patients according to IDO1 mRNA expression level in bone marrow specimens. IDO1-low group (n = 71) and IDO1-high group (n = 80) were compared using the log-rank test; (b) Forest plot of multivariant analysis of basic clinical information and IDO1 expression groups; (c) The correlations between IDO1 gene expression and relative proportions of 22 cell types in the BMM of AML patients from the TCGA LAML database using CIBERSORT method coupled with LM22. Fork marks indicate statistical significance below 0.05; (d) Scatter plots of the proportions of naïve (left), memory B cells (right) and IDO1 gene expression level (log2 (TPM+1)). Fig. S4. Flow cytometry gating strategy. (a) FCM gating strategy for detection of total B cells (CD19+) cells, naïve B cells (CD19+IgD+CD27-), memory B cells (CD19+CD27+), Breg cells (CD19+CD27+CD24+). (b) FCM gating strategy for detection of CD8+ T cells (CD45+CD3+CD8+). All gates were set using fluorescence-minus-one (FMO) controls. Fig. S5. IDO1 inhibitors significantly inhibited the upregulated [file 43556_2025_262_MOESM1_ESM.docx]

**Indoleamine 2,3-dioxygenase 1 Alters the Proportions of B Cell Subpopulations in the Microenvironment of Acute Myeloid Leukemia**

Yu Yao^1#^, Yu-ying Liu^1#^, Jian-feng Li^2^, Yun-shuo Chen^2^, Lei Shi^1^, Yang Shen^2^*, Li-li Yang^3^*, Qing Yang^1^*

^1^ State Key Laboratory of Genetics and Development of Complex Phenotypes, School of Life Sciences, Fudan University, Songhu Road 2005, Shanghai, 200438, China

^2^ Shanghai Institute of Hematology, State Key Laboratory of Medical Genomics, National Research Center for Translational Medicine at Shanghai, Ruijin Hospital Affiliated to Shanghai Jiao Tong University School of Medicine, Shanghai, 200025, China

^3^ Tianjin Medical University Cancer Institute and Hospital, National Clinical Research Center for Cancer, State Key Laboratory of Druggability Evaluation and Systematic Translational Medicine, Tianjin, 300060, China

*Corresponding authors:

Yang Shen, address: Shanghai Institute of Hematology, State Key Laboratory of Medical Genomics, National Research Center for Translational Medicine at Shanghai, Ruijin Hospital Affiliated to Shanghai Jiao Tong University School of Medicine, Shanghai, 200025, China, E-mail: [yang_shen@sjtu.edu.cn](mailto:yang_shen@sjtu.edu.cn).

Li-li Yang, address: Tianjin Medical University Cancer Institute and Hospital, National Clinical Research Center for Cancer, State Key Laboratory of Druggability Evaluation and Systematic Translational Medicine, Tianjin, 300060, China, E-mail: [yanglili@tjmuch.com](mailto:yanglili@tjmuch.com).

Qing Yang, address: State Key Laboratory of Genetics and Development of Complex Phenotypes, School of Life Sciences, Fudan University, Songhu Road 2005, Shanghai, 200438, China, telephone & fax number: +86-021-31246641, E-mail: yangqing68@fudan.edu.cn.

# These authors contributed equally to this work and should be considered co-first authors.

These supplementary materials contain specific data results that support the paper and provide researchers with a more complete picture of the details of our work.

**Supplementary Materials**

**Supplementary Figures**


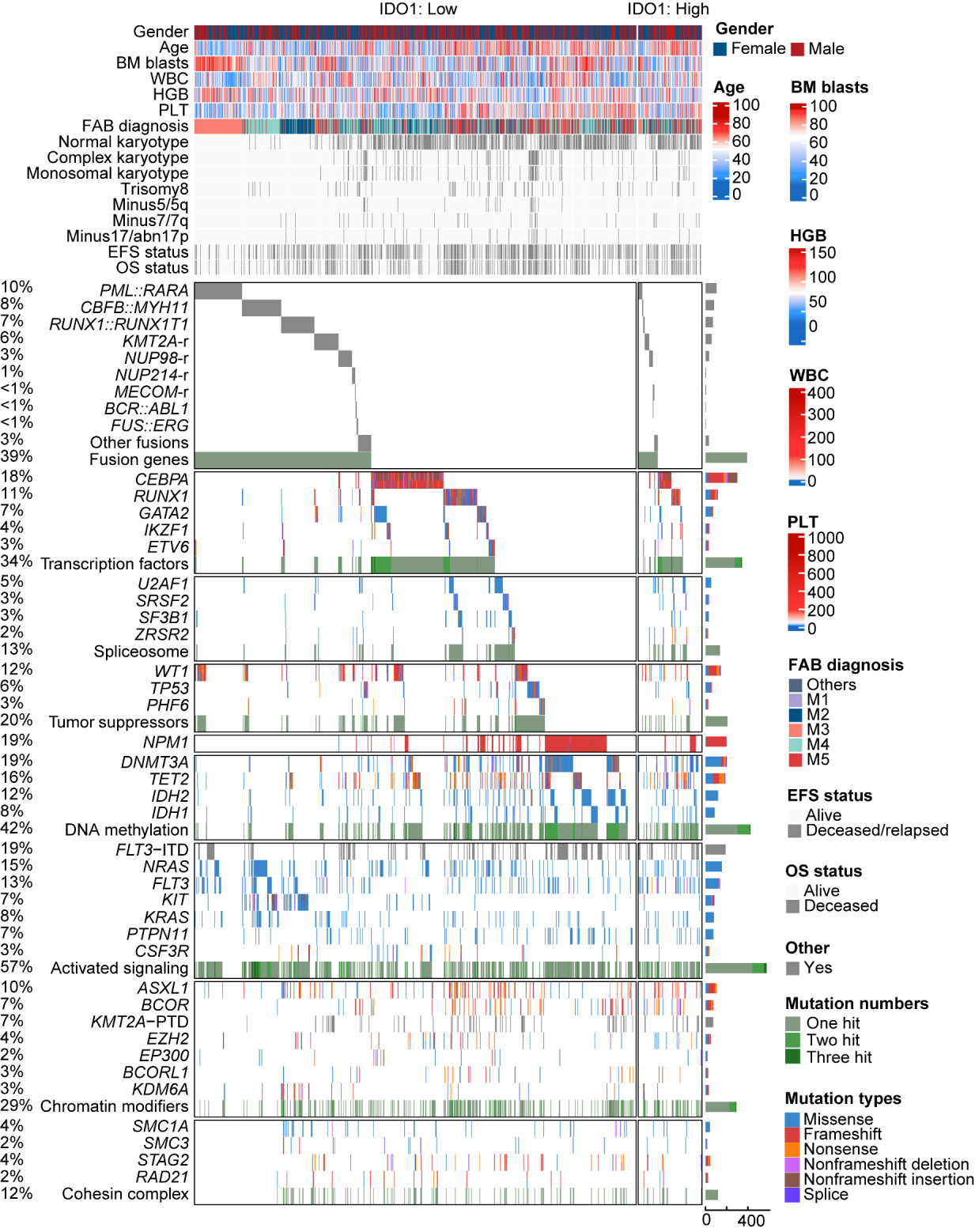


**Fig. S1 Differential gene comparison between** ***IDO1*-low and high groups of AML patients from Ruijin hospital.**

Each row represents a clinical or genomic characteristic. Each column represents one patient. The patients are divided into left (low) and right (high) panels according to the gene expression level of IDO1. The cutoff of low and high is determined by the prognosis significance. The right percentages and bar plots show the overall mutation rate and the number of mutations (genes or pathways).


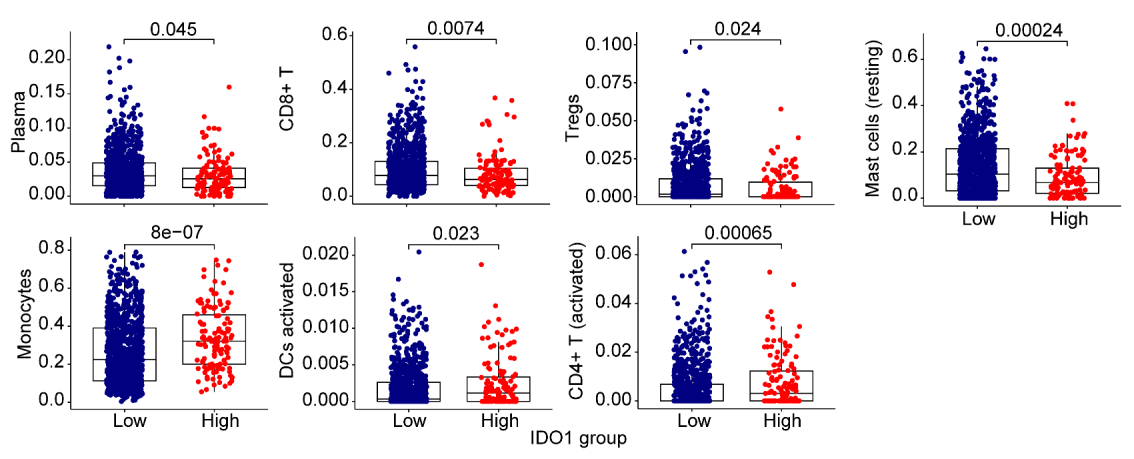


**Fig. S2 Other significant difference in immune fractions between** ***IDO1*-low and high groups of AML patients from Ruijin hospital.**

The statistical significance is calculated based on the Wilcoxon Rank Sum Test.


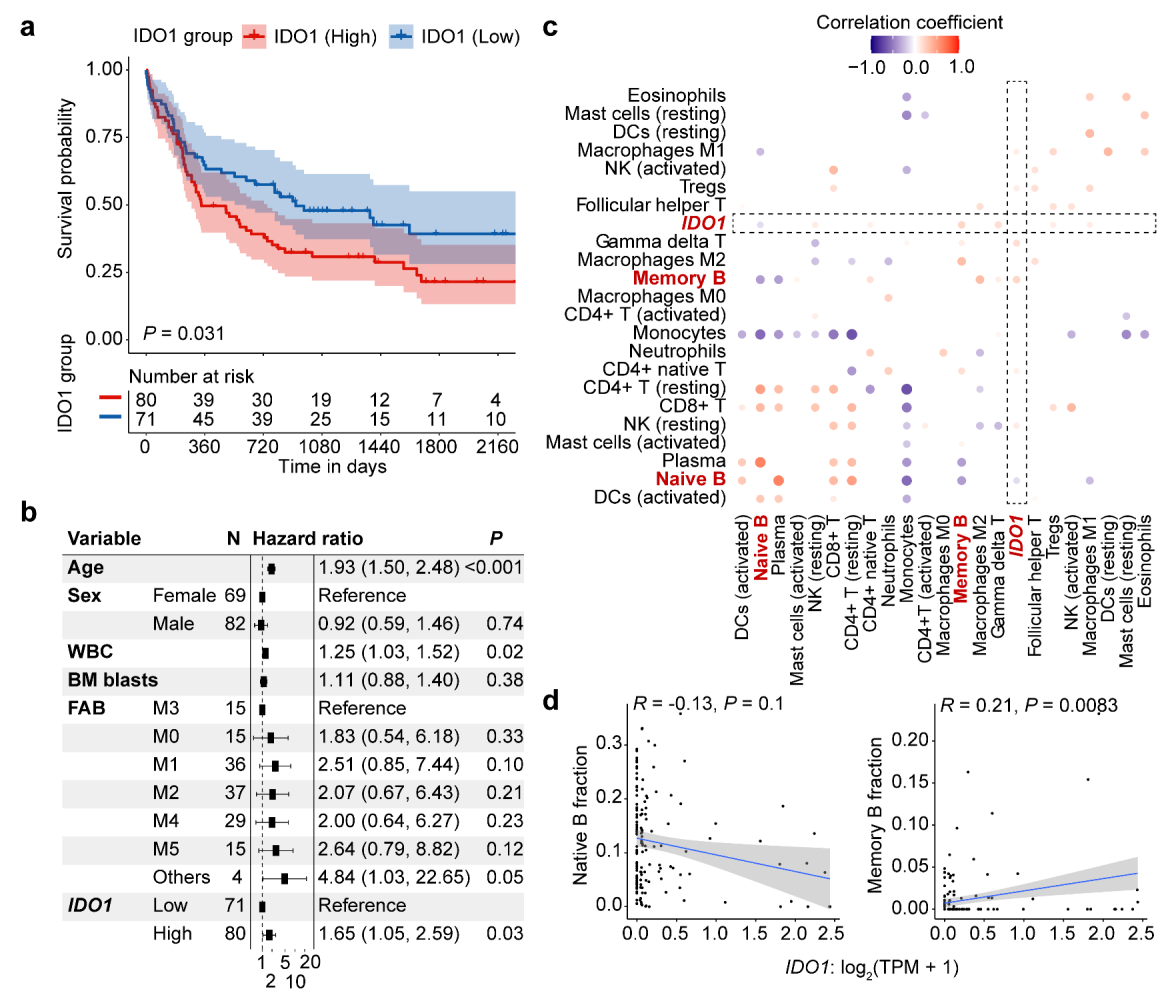


**Fig. S3 Correlations between *IDO1* gene expression and clinical prognosis, and between *IDO1* gene expression and proportions of naïve /memory B cells in the TCGA LAML cohort.**

(a) Kaplan-Meier survival curves of OS of the AML patients according to *IDO1* mRNA expression level in bone marrow specimens. IDO1-low group (n = 71) and IDO1-high group (n = 80) were compared using the log-rank test; (b) Forest plot of multivariant analysis of basic clinical information and *IDO1* expression groups; (c) The correlations between *IDO1* gene expression and relative proportions of 22 cell types in the BMM of AML patients from the TCGA LAML database using CIBERSORT method coupled with LM22. Fork marks indicate statistical significance below 0.05; (d) Scatter plots of the proportions of naïve (left), memory B cells (right) and *IDO1* gene expression level (log2 (TPM+1)).


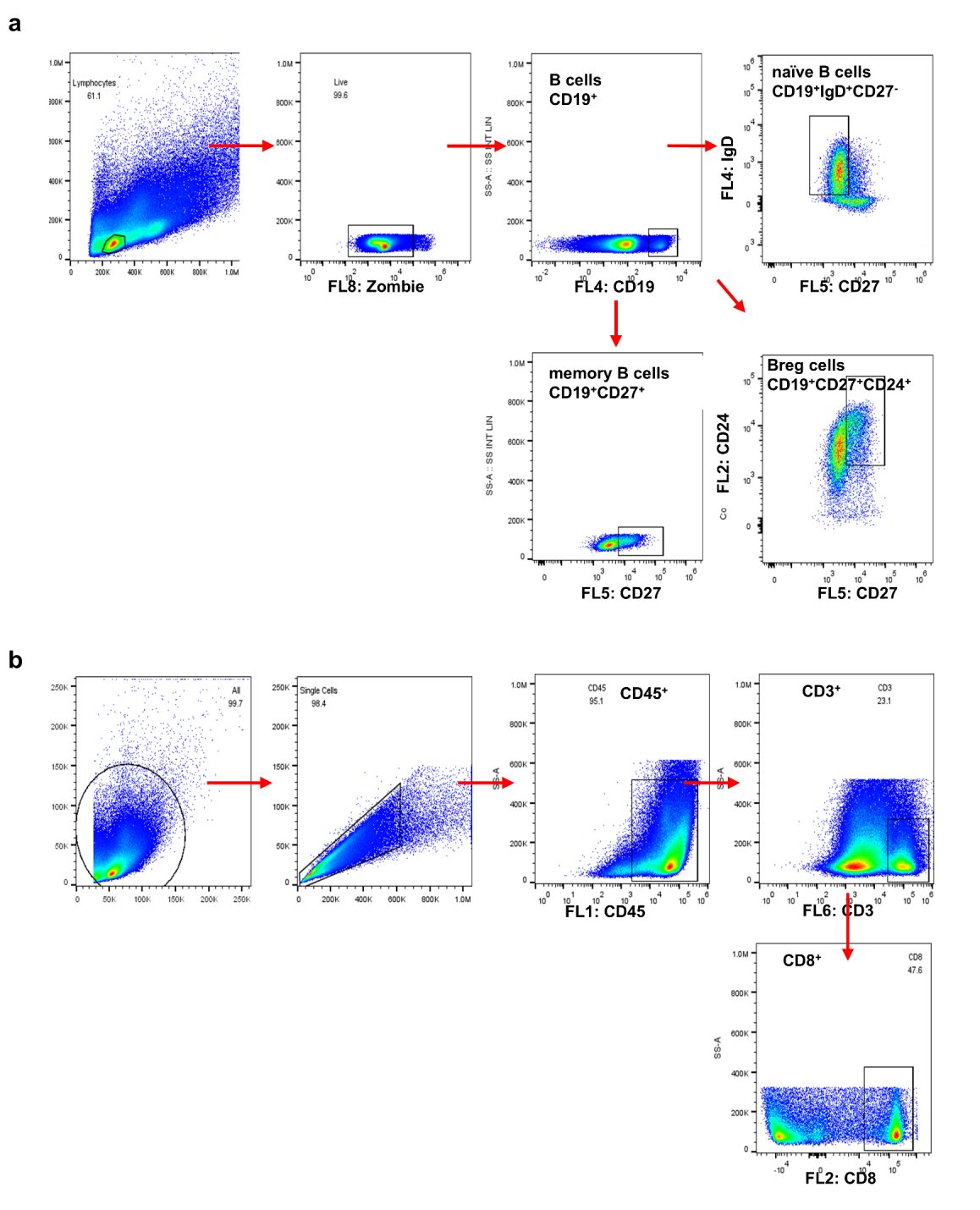


**Fig. S4 Flow cytometry gating strategy.**

(a) FCM gating strategy for detection of total B cells (CD19^+^) cells, naïve B cells (CD19^+^IgD^+^CD27^-^), memory B cells (CD19^+^CD27^+^), Breg cells (CD19^+^CD27^+^CD24^+^). (b) FCM gating strategy for detection of CD8^+^ T cells (CD45^+^ CD3^+^CD8^+^). All gates were set using fluorescence-minus-one (FMO) controls.


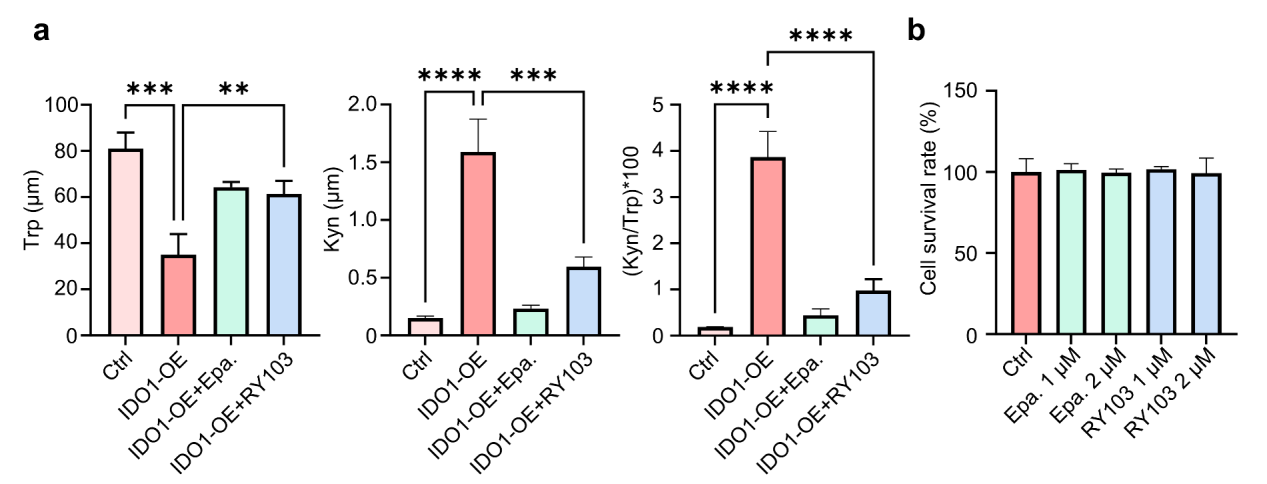


**Fig. S5 IDO1 inhibitor significantly inhibited the upregulated KP without cytotoxicity in hIDO1-OE OA3 cells.**

(a) HPLC analysis of Trp and Kyn levels in medium from OA3 cells (Ctrl), hIDO1-OE OA3 cells and IDO1 inhibitor (2 μM) treated hIDO1-OE OA3 cells. (b) CCK-8 analysis of cytotoxicity of IDO1 inhibitor on hIDO1-OE OA3 cells (Ctrl). Epa. represented epacadostat. Statistical significance was determined by one-way ANOVA followed by Dunnett’s post hoc test. Data were presented as the mean ± SD. * *p* < 0.05, ** *p* < 0.01, *** *p* < 0.001.


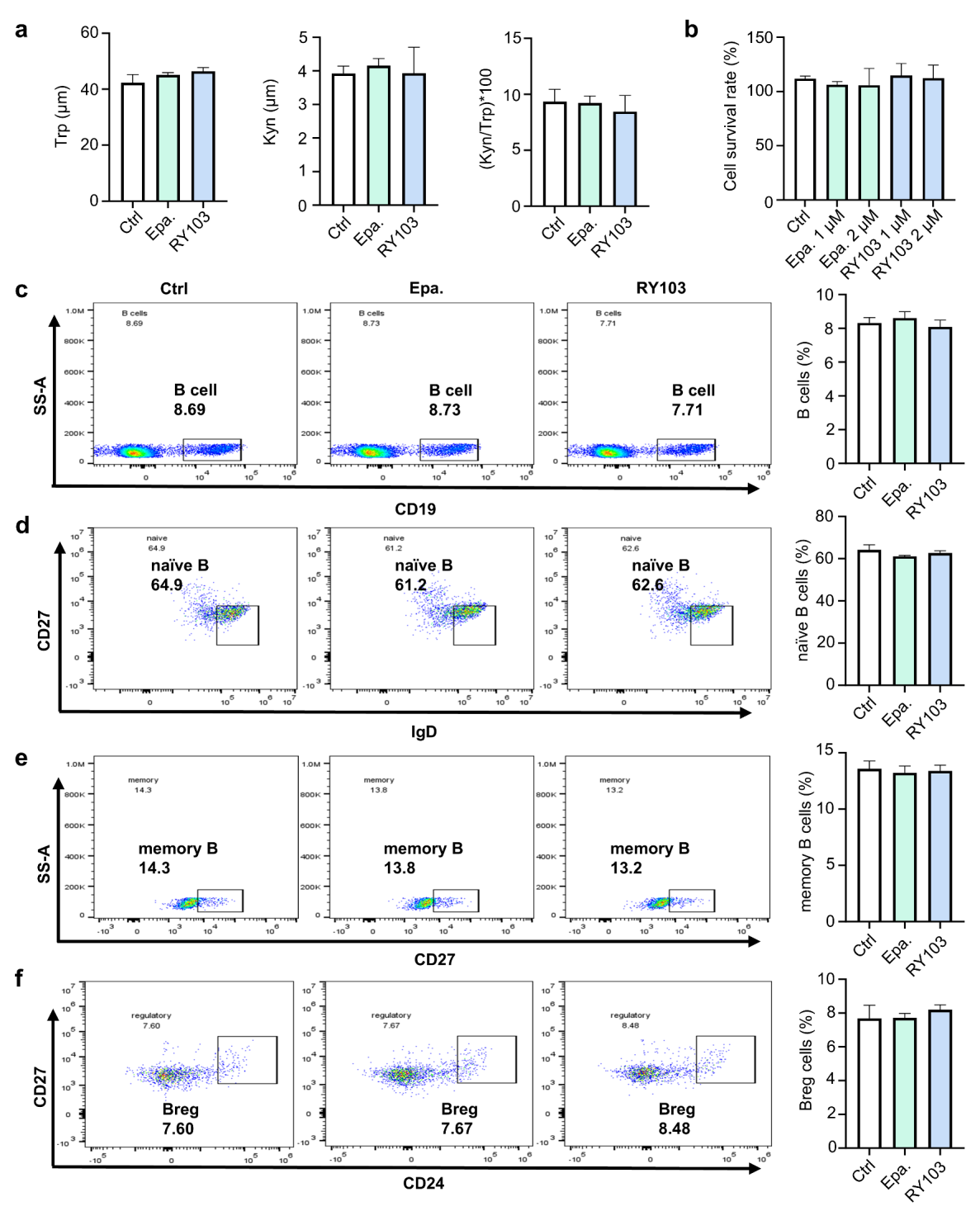


**Fig. S6** **IDO1 inhibitor did not affect the basal KP and the proportions of B cell subpopulations without cytotoxicity in healthy human PBMCs.**

PBMCs were treated with IDO1 inhibitor (2 μM unless stated otherwise) stimulated by IL-4 (10 ng/mL) and LPS (20 μg/mL) for 72 h, Epa. represented epacadostat. (a) HPLC analysis of Trp and Kyn levels in medium. (b) CCK-8 analysis of the viability of PBMCs. (c-f) Flow cytometry analysis of the proportions of total B cells (CD19^+^), naïve B cells (CD19^+^IgD^+^CD27^-^), memory B cells (CD19^+^CD27^+^) and Breg cells (CD19^+^CD24^+^CD27^+^).


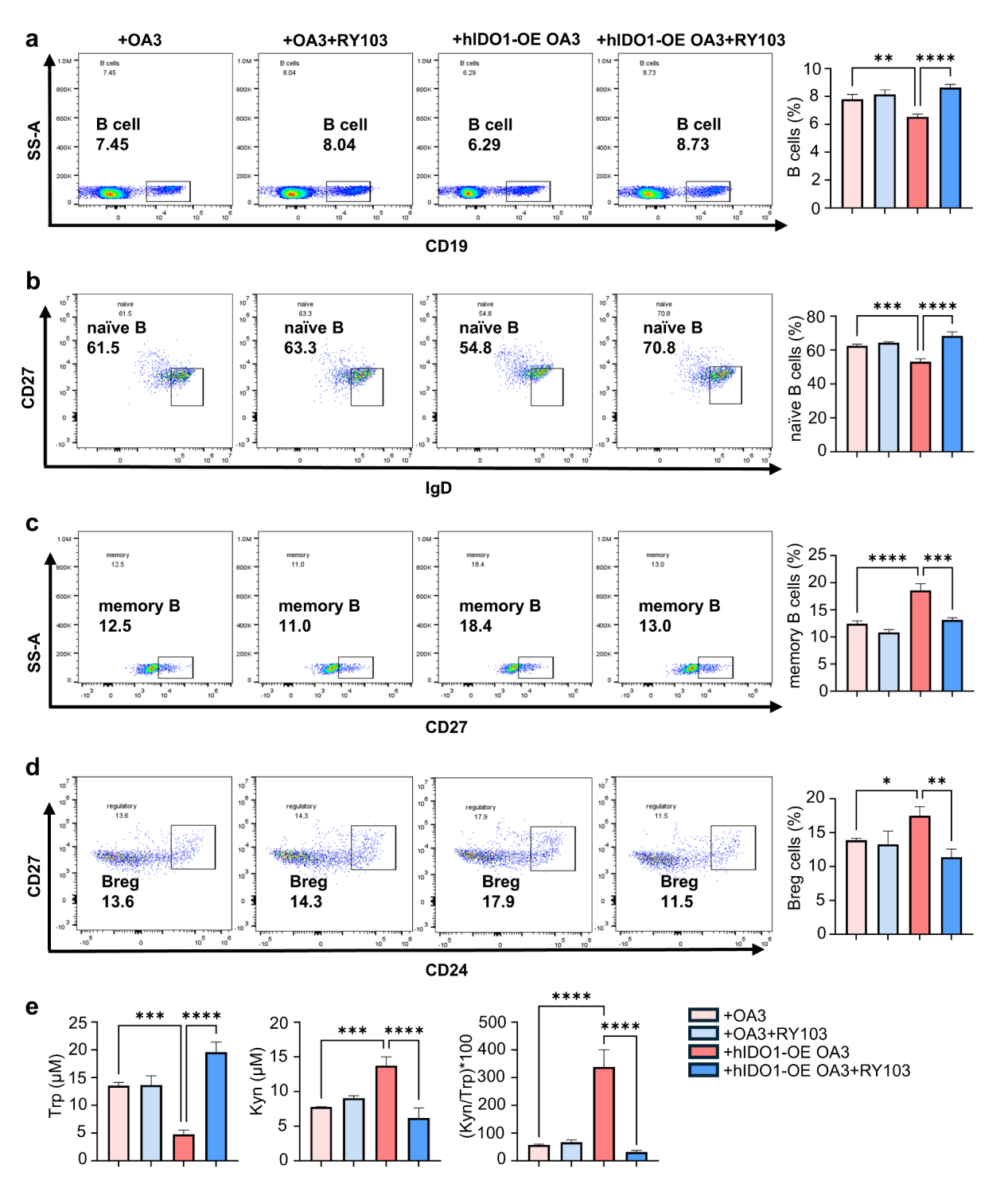


**Fig. S7 RY103 only changed the proportions of B cell subpopulations and KP activity in the co-culture of PBMCs and hIDO1-OE OA3 cells.**

PBMCs were co-cultured with OA3 cells or hIDO1-OE OA3 cells stimulated by IL-4 (10 ng/mL) and LPS (20 μg/mL), in the absence or presence of RY103 (2 μM) for 72 h. (a-d) Flow cytometry analysis of the proportions of total B cells (CD19^+^), naïve B cells (CD19^+^IgD^+^CD27^-^), memory B cells (CD19^+^CD27^+^) and Breg cells (CD19^+^CD24^+^CD27^+^). Representative histograms and quantification were shown. n=3 per group. (e) HPLC analysis of Trp and Kyn levels in medium. Statistical significance was determined by one-way ANOVA followed by Dunnett’s post hoc test for comparison. Data were presented as the mean ± SD. * *p* < 0.05, ** *p* < 0.01, *** *p* < 0.001.


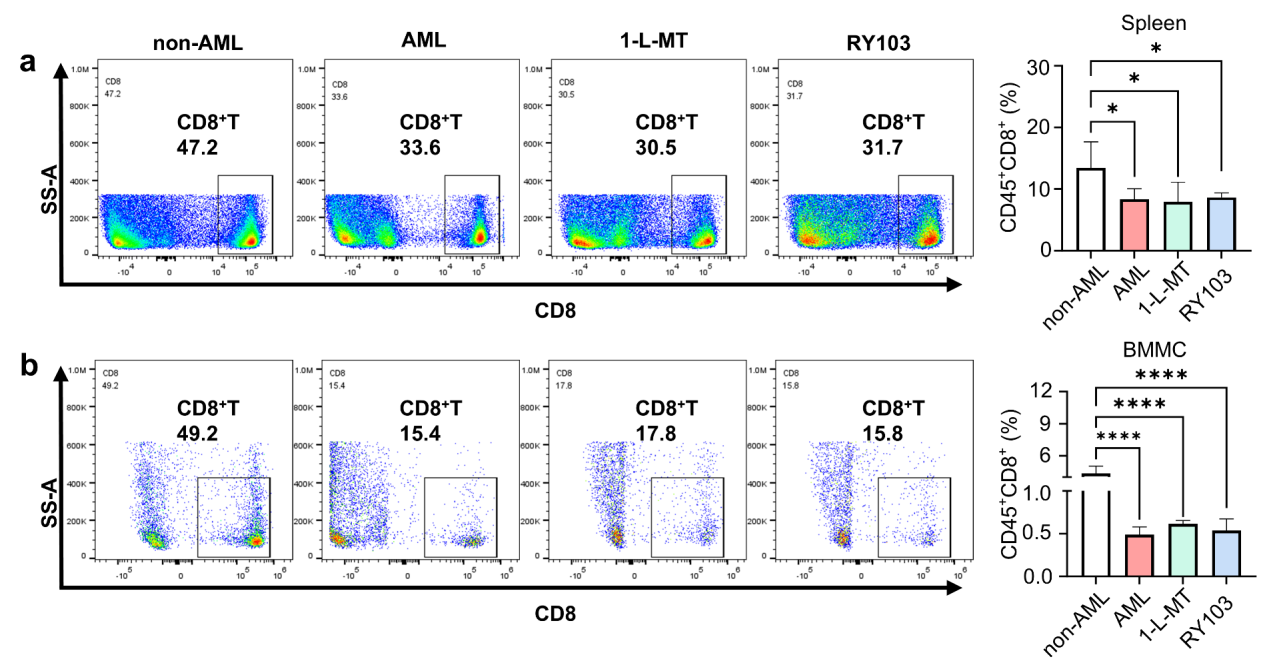


**Fig. S8 IDO1 inhibitor did not alter the proportion of CD8^+^ T cells in the spleen and bone marrow of AML mice.**

The construction of mouse models and the assignment of the different treatments were described in the Materials and Methods section. (a-b) Flow cytometry analysis of the proportion of CD8^+^T cells (CD45^+^CD8^+^) in spleen and BMMCs from different groups, n=5 per group. Representative histograms and quantification were shown. Statistical significance was determined by one-way ANOVA followed by Dunnett’s post hoc test. Data were presented as the mean ± SD. **p* < 0.05, ***p* < 0.01, ****p* < 0.001.

**Supplementary Tables**

**Table S1 Clinical characteristics of 5 AML patients from Ruijin Hospital included in this study**

| **Patient** | **Age** | **Gender** | **AML subtype** |
| --- | --- | --- | --- |
| 1 | 29 | Male | AML-M2 |
| 2 | 48 | Female | AML-M2b |
| 3 | 52 | Male | AML-M5b |
| 4 | 40 | Female | AML-M4 |
| 5 | 38 | Male | AML-M4 |

**Table S2 PCR Primers for quantification**

| **Primer** | **Sequences (5′-3′)** |
| --- | --- |
| *Human-β-actin* | Forward: GTTGTCGACGACGAGCG  Reverse: GCACAGAGCCTCGCCTT |
| *Human-IL-10* | Forward: TCTGGTGAAGGAGGATCGCTA  Reverse: GTTCTCAGCTTGGGGCATCA |
| *Human-TNF-α* | Forward: CTCTTCTGCCTGCTGCACTTTG  Reverse: ATGGGCTACAGGCTTGTCACTC |
| *Human-IL-35* | Forward: TTACAAGCGTCAGGGAGCTG  Reverse: TTCCCCGTAGTCTGTGAGGT |
| *Human-PIK3CA* | Forward: CCACGACCATCATCAGGTGAA  Reverse: CCTCACGGAGGCATTCTAAAGT |
| *Human-PIK3R1* | Forward: TGGACGGCGAAGTAAAGCATT  Reverse: AGTGTGACATTGAGGGAGTCG |
| *Human-PDK1* | Forward: GAGAGCCACTATGGAACACCA  Reverse: GGAGGTCTCAACACGAGGT |
| *Human-Akt* | Forward: AATGGACAGAAGCTATCCAGGC  Reverse: TGATGGGTTGTAGAGGCATCC |
| *Mouse-β-actin* | Forward: ACTCCTATGTGGGTGACGAGG  Reverse: TTTTCACGGTTGGCCTTAGGGTT |
| *Mouse-IL-10* | Forward: AGGCGCTGTCATCGATTTCT  Reverse: GGCCTTGTAGACACCTTGGTC |
| *Mouse-TNF-α* | Forward: ACCCTCACACTCACAAACCA  Reverse: ACAAGGTACAACCCATCGGC |
| *Mouse-IL-35* | Forward: AGAGTGCAATGCCATGCTTC  Reverse: CCGAGCCTGTAAGTGGCAAT |

**Table S3 Clinical and molecular features in IDO1-low and high groups of AML patients from Ruijin hospital.**

| **Variable** | **High**, N = 127^1^ | **Low**, N = 880^1^ | **p-value**^2^ |
| --- | --- | --- | --- |
| **Gender** |  |  | **0.051** |
| Female | 50 (39) | 428 (49) |  |
| Male | 77 (61) | 452 (51) |  |
| **Age** | 56 (44 – 66) | 51 (38 – 62) | **0.002** |
| **BM blasts** | 59 (38 – 74) | 67 (46 – 85) | **0.002** |
| **WBC** | 14 (4 – 49) | 10 (3 – 40) | **0.030** |
| NA | 10 | 38 |  |
| **HGB** | 76 (63 – 98) | 82 (66 – 101) | **0.089** |
| NA | 10 | 38 |  |
| **PLT** | 43 (20 – 85) | 41 (22 – 83) | 0.56 |
| NA | 10 | 38 |  |
| **FAB diagnosis** |  |  | 0.54 |
| M1 | 2 (1.6) | 18 (2.0) |  |
| M2 | 11 (8.7) | 99 (11) |  |
| M3 | 8 (6.3) | 95 (11) |  |
| M4 | 47 (37) | 315 (36) |  |
| M5 | 35 (28) | 203 (23) |  |
| Others | 24 (19) | 150 (17) |  |
| **Normal karyotype** | 63 (51) | 357 (41) | **0.035** |
| NA | 4 | 13 |  |
| **Complex karyotype** | 8 (6.5) | 69 (8.0) | 0.57 |
| NA | 4 | 13 |  |
| **Monosomal karyotype** | 9 (7.3) | 63 (7.3) | 0.98 |
| NA | 4 | 13 |  |
| **Trisomy8** | 7 (5.7) | 52 (6.0) | 0.89 |
| NA | 4 | 13 |  |
| **Minus5/5q** | 0 (0) | 19 (2.2) | 0.15 |
| NA | 4 | 13 |  |
| **Minus7/7q** | 7 (5.7) | 25 (2.9) | 0.10 |
| NA | 4 | 13 |  |
| **Minus17/abn17p** | 2 (1.6) | 25 (2.9) | 0.56 |
| NA | 4 | 13 |  |
| ***PML::RARA*** | 8 (6.3) | 95 (11) | **0.12** |
| ***CBFB::MYH11*** | 2 (1.6) | 78 (8.9) | **0.005** |
| ***RUNX1::RUNX1T1*** | 3 (2.4) | 66 (7.5) | **0.032** |
| ***KMT2A*-r** | 9 (7.1) | 48 (5.5) | 0.46 |
| ***NUP98*-r** | 7 (5.5) | 27 (3.1) | 0.18 |
| ***NUP214*-r** | 0 (0) | 6 (0.7) | >0.99 |
| ***MECOM*-r** | 3 (2.4) | 1 (0.1) | **0.007** |
| ***BCR::ABL1*** | 1 (0.8) | 2 (0.2) | 0.33 |
| ***FUS::ERG*** | 0 (0) | 3 (0.3) | >0.99 |
| **Other fusions** |  |  | **0.012** |
| **Fusion negative** | 88 (69) | 528 (60) |  |
| **No** | 32 (25) | 326 (37) |  |
| **Yes** | 7 (5.5) | 26 (3.0) |  |
| **Fusion genes** | 39 (31) | 352 (40) | **0.045** |
| ***CEBPA*** | 30 (24) | 150 (17) | **0.071** |
| ***RUNX1*** | 23 (18) | 85 (9.7) | **0.004** |
| ***GATA2*** | 9 (7.1) | 59 (6.7) | 0.87 |
| ***IKZF1*** | 7 (5.5) | 29 (3.3) | 0.20 |
| ***ETV6*** | 6 (4.7) | 23 (2.6) | 0.25 |
| **Transcription factors** | 59 (46) | 284 (32) | **0.002** |
| ***U2AF1*** | 13 (10) | 40 (4.5) | **0.007** |
| ***SRSF2*** | 6 (4.7) | 27 (3.1) | 0.29 |
| ***SF3B1*** | 4 (3.1) | 26 (3.0) | 0.78 |
| ***ZRSR2*** | 6 (4.7) | 15 (1.7) | **0.039** |
| **Spliceosome** | 28 (22) | 106 (12) | **0.002** |
| ***WT1*** | 14 (11) | 111 (13) | 0.61 |
| ***TP53*** | 6 (4.7) | 50 (5.7) | 0.66 |
| ***PHF6*** | 2 (1.6) | 26 (3.0) | 0.56 |
| **Tumor suppressors** | 22 (17) | 182 (21) | 0.38 |
| ***NPM1*** | 16 (13) | 180 (20) | **0.037** |
| ***DNMT3A*** | 28 (22) | 162 (18) | 0.33 |
| ***TET2*** | 28 (22) | 130 (15) | **0.035** |
| ***IDH2*** | 11 (8.7) | 108 (12) | 0.24 |
| ***IDH1*** | 10 (7.9) | 74 (8.4) | 0.84 |
| **DNA methylation** | 60 (47) | 364 (41) | 0.21 |
| ***FLT3*-ITD** | 27 (21) | 162 (18) | 0.44 |
| ***NRAS*** | 17 (13) | 136 (15) | 0.54 |
| ***FLT3*** | 27 (21) | 106 (12) | **0.004** |
| ***KIT*** | 2 (1.6) | 72 (8.2) | **0.008** |
| ***KRAS*** | 9 (7.1) | 68 (7.7) | 0.80 |
| ***PTPN11*** | 12 (9.4) | 62 (7.0) | 0.33 |
| ***CSF3R*** | 1 (0.8) | 34 (3.9) | 0.11 |
| **Activated signaling** | 70 (55) | 506 (58) | 0.61 |
| ***ASXL1*** | 15 (12) | 87 (9.9) | 0.50 |
| ***BCOR*** | 14 (11) | 57 (6.5) | **0.061** |
| ***KMT2A-*PTD** | 11 (8.7) | 62 (7.0) | 0.51 |
| ***EZH2*** | 8 (6.3) | 35 (4.0) | 0.23 |
| ***EP300*** | 2 (1.6) | 16 (1.8) | >0.99 |
| ***BCORL1*** | 3 (2.4) | 23 (2.6) | >0.99 |
| ***KDM6A*** | 4 (3.1) | 27 (3.1) | >0.99 |
| **Chromatin modifiers** | 42 (33) | 250 (28) | 0.28 |
| ***SMC1A*** | 4 (3.1) | 36 (4.1) | 0.61 |
| ***SMC3*** | 3 (2.4) | 14 (1.6) | 0.46 |
| ***STAG2*** | 5 (3.9) | 37 (4.2) | 0.89 |
| ***RAD21*** | 3 (2.4) | 19 (2.2) | 0.75 |
| **Cohesin Complex** | 14 (11) | 102 (12) | 0.85 |
| ^1^n (%); Median (IQR) | | | |
| ^2^Pearson's Chi-squared test; Wilcoxon rank sum test; Fisher's exact test  p-values in bold represented genes mentioned in the main text. | | | |
