# Supplementary material for: Indoleamine 2,3-dioxygenase 1 alters the proportions of B cell subpopulations in the microenvironment of acute myeloid leukemia
Source: Mol Biomed. 2025 Apr 16;6:23. doi: 10.1186/s43556-025-00262-x (PMC12000501; doi:10.1186/s43556-025-00262-x)
Supplement: Supplementary file 2 — Supplementary Material 2. [file 43556_2025_262_MOESM2_ESM.docx]

**Indoleamine 2,3-dioxygenase 1 Alters the Proportions of B Cell Subpopulations in the Microenvironment of Acute Myeloid Leukemia**

Yu Yao^1#^, Yu-ying Liu^1#^, Jian-feng Li^2^, Yun-shuo Chen^2^, Lei Shi^1^, Yang Shen^2^*, Li-li Yang^3^*, Qing Yang^1^*

^1^ State Key Laboratory of Genetics and Development of Complex Phenotypes, School of Life Sciences, Fudan University, Songhu Road 2005, Shanghai, 200438, China

^2^ Shanghai Institute of Hematology, State Key Laboratory of Medical Genomics, National Research Center for Translational Medicine at Shanghai, Ruijin Hospital Affiliated to Shanghai Jiao Tong University School of Medicine, Shanghai, 200025, China

^3^ Tianjin Medical University Cancer Institute and Hospital, National Clinical Research Center for Cancer, State Key Laboratory of Druggability Evaluation and Systematic Translational Medicine, Tianjin, 300060, China

*Corresponding authors:

Yang Shen, address: Shanghai Institute of Hematology, State Key Laboratory of Medical Genomics, National Research Center for Translational Medicine at Shanghai, Ruijin Hospital Affiliated to Shanghai Jiao Tong University School of Medicine, Shanghai, 200025, China, E-mail: [yang_shen@sjtu.edu.cn](mailto:yang_shen@sjtu.edu.cn).

Li-li Yang, address: Tianjin Medical University Cancer Institute and Hospital, National Clinical Research Center for Cancer, State Key Laboratory of Druggability Evaluation and Systematic Translational Medicine, Tianjin, 300060, China, E-mail: [yanglili@tjmuch.com](mailto:yanglili@tjmuch.com).

Qing Yang, address: State Key Laboratory of Genetics and Development of Complex Phenotypes, School of Life Sciences, Fudan University, Songhu Road 2005, Shanghai, 200438, China, telephone & fax number: +86-021-31246641, E-mail: yangqing68@fudan.edu.cn.

# These authors contributed equally to this work and should be considered co-first authors.

These supplementary materials contain specific data results that support the paper and provide researchers with a more complete picture of the details of our work.

**Supplementary Materials**

**
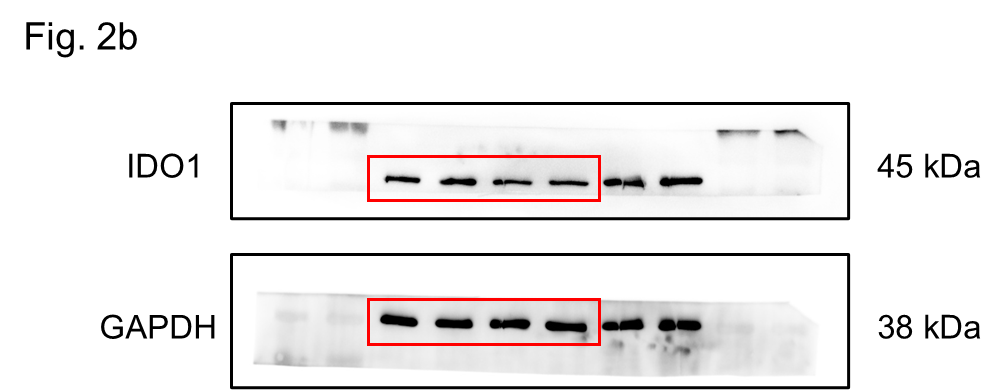
**

**
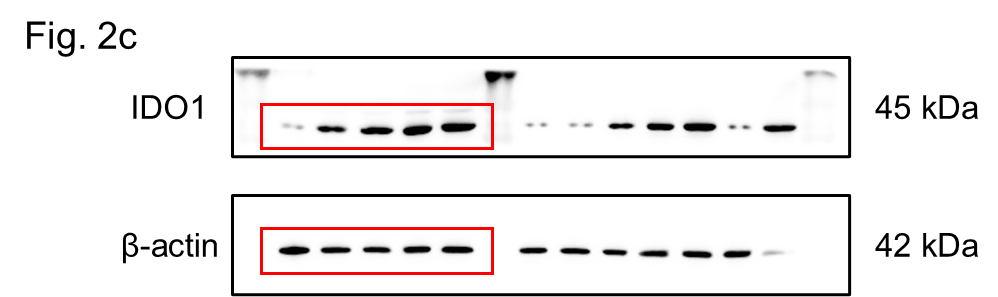
**

**
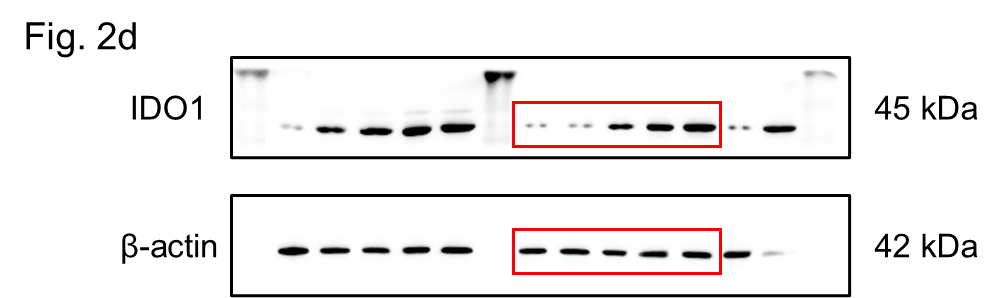
**

**
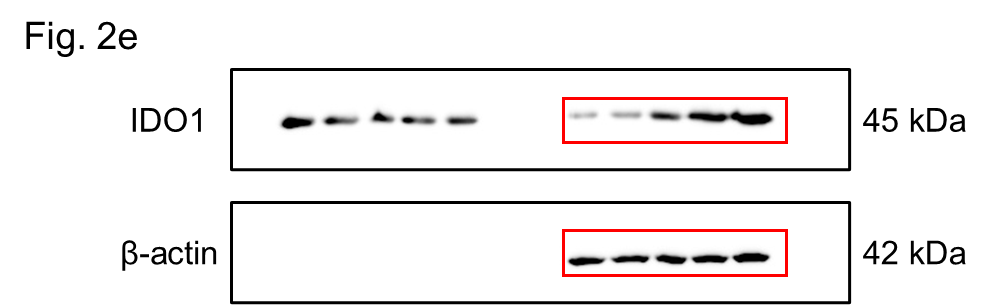
**

**
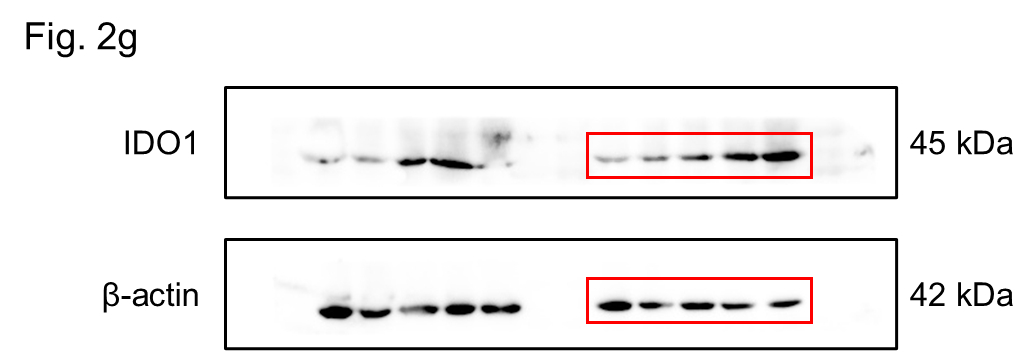
**

**
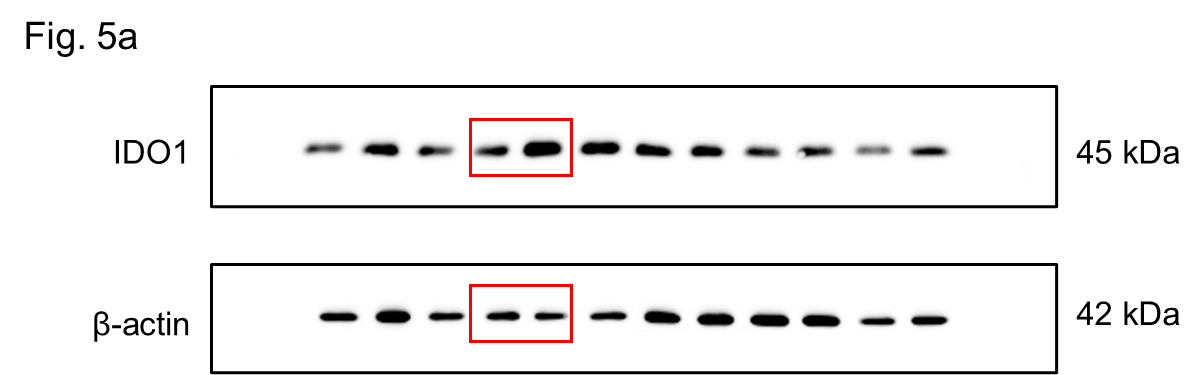
**
